# Comparative Efficacy and Safety of Potassium-Competitive Acid Blockers vs. Proton Pump Inhibitors for Peptic Ulcer with or without *Helicobacter pylori* Infection: A Systematic Review and Network Meta-Analysis

**DOI:** 10.3390/ph17060698

**Published:** 2024-05-28

**Authors:** Mengling Ouyang, Shupeng Zou, Qian Cheng, Xuan Shi, Yazheng Zhao, Minghui Sun

**Affiliations:** Department of Pharmacy, Tongji Hospital, Tongji Medical College, Huazhong University of Science and Technology, Wuhan 430000, China; oyml@hust.edu.cn (M.O.); tjzoushupeng@tjh.tjmu.edu.cn (S.Z.); chengqian0713@tjh.tjmu.edu.cn (Q.C.); xuanshi@tjh.tjmu.edu.cn (X.S.); yazh_zhao@tjh.tjmu.edu.cn (Y.Z.)

**Keywords:** potassium-competitive acid blocker, proton pump inhibitor, peptic ulcer, *Helicobacter pylori*, network meta-analysis

## Abstract

Novel potassium-competitive acid blockers (P-CABs) have emerged as effective acid-suppressive drugs in recent years, replacing proton pump inhibitors (PPIs). We aim to compare the efficacy and safety of P-CABs versus PPIs in the treatment of peptic ulcers with or without *Helicobacter pylori* (*H. pylori*) infection. We searched in PubMed, Embase, WOS, Cochrane Library, ClinicalTrials.gov, CNKI, and Wanfang databases (all years up to January 2024). Efficacy and safety outcomes were evaluated using odds ratio (OR) and 95% confidence intervals (CI). The Surface Under the Cumulative Ranking (SUCRA) probabilities were used to rank each intervention. Among 14,056 studies screened, 56 studies involving 9792 participants were analyzed. Vonoprazan demonstrated the best efficacy in ulcer healing rate and *H. pylori* eradication rate (SUCRA = 86.4% and 90.7%, respectively). Keverprazan ranked second in ulcer healing rates (SUCRA = 76.0%) and was more effective in pain remission rates (SUCRA = 91.7%). The risk of adverse events was low for keverprazan (SUCRA = 11.8%) and tegoprazan (SUCRA = 12.9%), and moderate risk for vonoprazan (SUCRA = 44.3%) was demonstrated. Compared to lansoprazole, vonoprazan exhibited a higher risk of drug-related adverse events (OR: 2.15; 95% CI: 1.60–2.89) and serious adverse events (OR: 2.22; 95% CI: 1.11–4.42). Subgroup analysis on patients with *H. pylori*-positive peptic ulcers showed that vonoprazan was at the top of the SUCRA rankings, followed by keverprazan. Vonoprazan showed superior performance in peptic ulcers, especially for patients with *H. pylori*-positive peptic ulcers. However, the risk of adverse events associated with vonoprazan should be noted. Keverprazan has also shown good therapeutic outcomes and has performed better in terms of safety.

## 1. Introduction

Peptic ulcer is a chronic acid-related disease, mainly manifested by recurrent abdominal pain and bloating, accompanied by vomiting, acid reflux, and abnormal stool, commonly found in the stomach and duodenum. It is estimated that the lifetime prevalence of peptic ulcers in the general population is about 5–10%, with an annual incidence of 0.1–0.3% [1]. The pathogenesis of peptic ulcer is the imbalance between the invasion of gastric acid and pepsin and the defense and repair capacity of the mucosa, leading to self-digestion of the mucosa by gastric acid [2]. *Helicobacter pylori* (*H. pylori*) infection and the use of non-steroidal anti-inflammatory drugs (NSAIDs) are the main causative factors for peptic ulcer [3].

At present, the main treatment options for peptic ulcer include acid-suppressive drugs, gastric mucosal protection agents, anti-*H. pylori* therapy, and discontinuation of NSAIDs [4,5]. Acid suppressive drug therapy is the most commonly used treatment method, including the use of proton pump inhibitors (PPIs), potassium-competitive acid blockers (P-CABs), and H_2_ receptor blockers (H2RAs). Previous studies have shown that PPIs have a stronger acid inhibition effect than H2RAs, can more effectively control the symptoms of peptic ulcer, and improve ulcer healing rates [6]. However, PPIs have some shortcomings such as unstable acid inhibition under acidic conditions, slow onset of action, short duration of effect, and nocturnal acid breakthrough [7,8]. In addition, some studies have found that the use of PPIs is associated with diseases such as community-acquired pneumonia, Clostridium difficile infection, and chronic kidney disease [9,10,11].

P-CABs are a new type of acid-suppressive drug that can inhibit gastric acid secretion by competitively inhibiting the binding of resting and active state proton pumps to potassium ions and inhibiting the exchange of H+ and K+ [12]. P-CABs overcome the shortcomings of traditional PPIs and can produce potent and long-lasting acid-suppressing effects at the first administration, thus potentially becoming an alternative for acid-related diseases [13,14]. Currently, P-CABs, such as revaprazan, vonoprazan, tegoprazan, and keverprazan, have obtained clinical approval [15,16]. Previous studies have found that vonoprazan has some efficacy against refractory NSAID-induced ulcers that cannot be controlled by traditional PPIs [17].

Currently, there is a meta-analysis comparing the efficacy of P-CABs and lansoprazole in the treatment of peptic ulcer [18]. However, there is a lack of evidence-based medical evidence to compare different PPIs and P-CABs in the treatment of peptic ulcers. Network meta-analysis can use a common comparator to make indirect comparisons when head-to-head comparisons are not available and combine direct and indirect comparisons to compare multiple interventions simultaneously [19]. We conducted a systematic review of the literature and a network meta-analysis to assess the efficacy and safety of different PPIs, P-CABs, and placebos in patients with peptic ulcers and to further analyze the efficacy of peptic ulcers in patients with or without *H. pylori* infection.

## 2. Methods

### 2.1. Search Strategy and Data Sources

This network meta-analysis was pre-registered at PROSPERO (registration number: 42023444341) and according to the guidelines of Preferred Reporting Items for Systematic Reviews and Meta-Analyses (PRISMA) [20].

The literature was searched from PubMed, Embase, Web of Science (WOS), Cochrane Library, ClinicalTrials.gov, China National Knowledge Infrastructure (CNKI), and Wanfang databases (all years up to January 2024) and used the following keywords: potassium-competitive acid blocker, proton pump inhibitor, peptic ulcer, and their types. In addition, other data sources were considered, such as the possible references cited in included studies and relevant review articles.

### 2.2. Study Selection

We formulated the inclusion and exclusion criteria before database searching. The inclusion criteria were as follows: (a) Patients: patients who underwent peptic ulcer, including gastric or duodenal ulcer; (b) Intervention/comparator: P-CABs (vonoprazan, tegoprazan, revaprazan, keverprazan), PPIs (omeprazole, lansoprazole, pantoprazole, rabeprazole, esomeprazole, ilaprazole), and placebo; (c) Outcome: ulcer healing rates, *H. pylori* eradication rates, remission rate of ulcer-related symptoms, and adverse events; (d) Study: randomized controlled trials (RCTs). The exclusion criteria were as follows: (a) Incompatible interventions: studies that excluded comparisons between PPIs, H2RAs, gastric mucosal protection agents, etc.; use other antibiotics to eradicate *H. pylori*, such as metronidazole, levofloxacin, furazolidone; (b) Inconsistent randomization: by order of diagnosis, parity grouping, etc.; (c) Incompatible disease types: ulcers after endoscopic submucosal dissection, stress ulcers, refractory ulcers, giant ulcers, and recurrent ulcers; (d) Inconsistent study types: retrospective trials, animal studies, reviews, conference papers, master’s and doctoral theses, etc.; (e) Intragroup comparison of different doses; use injections.

Two researchers (QC and XS) independently screened titles and abstracts and reviewed the full text based on the inclusion and exclusion criteria from the databases. When a disagreement occurred, another investigator participated with others in the discussion of the disputed study until a consensus was reached.

### 2.3. Data Extraction and Outcomes

Two researchers (MO and SZ) designed the data extraction form and extracted the data independently. For each included study, the following information was extracted: study ID (first author and publication year), trial number, country, study design, medication of experimental group and control group, duration of treatment, and outcomes assessed. When necessary, missing data were added by emailing the corresponding author.

Two independent investigators (MO and YZ) evaluated the included studies using Cochrane’s Risk of Bias (ROB) tool 2.0 [21]. This tool assessed five bias domains: (1) Randomization process; (2) Deviation from intended intervention; (3) Missing outcome data; (4) Measurement of the outcome; (5) Selection of the reported result. The overall judgment of risk of bias was classified as low risk, some concerns, and high risk.

The primary efficacy endpoint was the ulcer healing rate of peptic ulcer, as confirmed by endoscopy. The secondary efficacy endpoints included *H. pylori* eradication rates and remission rates of ulcer-related gastrointestinal symptoms. Ulcer-related gastrointestinal symptoms included abdominal pain, abdominal distension, nausea/vomiting, heartburn, regurgitation, epigastric burning sensation, lack of appetite, and belching. Safety analysis was based on adverse events, adverse events of P-CABs or PPIs alone or in combination with antibiotics for *H. pylori* eradication, drug-related adverse events, and serious adverse events.

### 2.4. Subgroup Analysis

We performed several subgroup analyses based on *H. pylori* infection (negative or positive), ulcer location (duodenal ulcer or gastric ulcer), duration of treatment (<4 weeks or 4–6 weeks or >6 weeks), and continent (Asia or Europe or North America).

### 2.5. Statistical Analysis

In this network meta-analysis, to estimate effect sizes for categorical outcome indicators, we calculated the odds ratio (OR) with 95% confidence intervals (95% CI) using the Mantel-Haenszel method. If the OR (95% CI) includes 1, it indicates that there is no difference between the two groups. If the OR (95% CI) is greater than 1, it suggests that the odds of the event are higher in the experimental group compared to the control group. Conversely, it indicates that the odds of the event are lower in the experimental group compared to the control group. We performed global and local inconsistency tests using the interaction by treatment design (Wald test) and the node-splitting methods, respectively [22]. The surfaces under the cumulative ranking curve (SUCRA) were used to predict the efficacy or safety of each treatment regimen, and SUCRA graphs were plotted for each outcome index. The higher SUCRA values indicate higher efficacy or safety. Finally, the funnel plot was used to assess publication bias. In addition, the heterogeneity among studies was assessed using the I^2^ statistic; If I^2^ > 50%, a high degree of heterogeneity among studies was considered to exist. If there was a high heterogeneity, we used a random-effects model for network meta-analysis [23]. All the statistical processes and result visualizations were conducted by Stata (version 17.0). The *p* value < 0.05 was considered statistically significant.

## 3. Results

### 3.1. Characteristics of Studies

**Figure 1:**
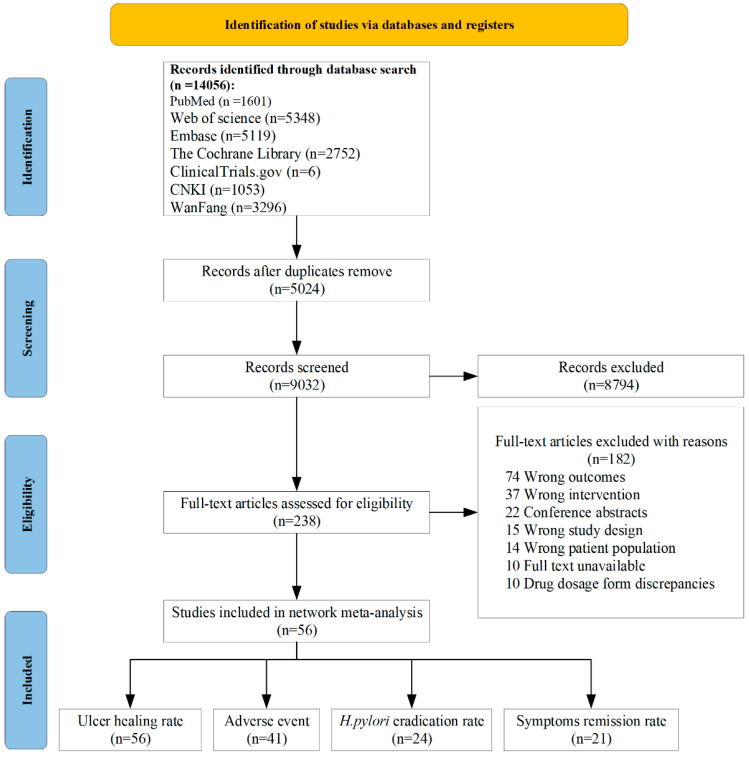
Flow diagram of study selection in the network meta-analysis. CNKI: China National Knowledge Infrastructure; *H. pylori*: *Helicobacter pylori*.

The process of study selection is depicted in Figure 1. Out of the 14,056 studies identified through the initial search, 56 RCTs, comprising 9792 patients, met all inclusion criteria and were deemed suitable for network meta-analysis [24,25,26,27,28,29,30,31,32,33,34,35,36,37,38,39,40,41,42,43,44,45,46,47,48,49,50,51,52,53,54,55,56,57,58,59,60,61,62,63,64,65,66,67,68,69,70,71,72,73,74,75,76,77]. Of the included RCTs, 44 were conducted in Asia, eight in Europe, and four in the USA. Among the included RCTs, 26 were single-center trials and 30 were multicenter clinical studies. These trials assessed three different P-CABs (vonoprazan, tegoprazan, and keverprazan), six different PPIs (omeprazole, lansoprazole, pantoprazole, rabeprazole, esomeprazole, ilaprazole) and placebo. Of the peptic ulcer cases, 28 were duodenal ulcers, 10 were gastric ulcers, six studies simultaneously evaluated both duodenal and gastric ulcers, and 12 were unspecified with regard to specific sites. A total of 56 RCTs reported outcomes related to ulcer healing rates, 24 focused on *H. pylori* eradication rate, 21 examined the remission rate of ulcer-related symptoms, and 41 documented adverse events. Out of the 56 included studies, 45 provided specific date ranges. The earliest studies were conducted from February 1990 to August 1991, while the most recent studies span from January 2020 to December 2022. The detailed characteristics of the included studies are shown in Tables S3–S6.

### 3.2. Quality Assessment

Overall, the risk of bias was generally low-to-some concerns. Out of the 56 included RCTs, 30 studies (53.6%) were deemed to have low risks of bias and 23 studies (41.1%) had some concerns due to not reporting the randomization and concealment of the allocation process. Additionally, three studies (5.4%) were considered to have high risks of bias due to issues with randomization and outcome measures. Insufficient details regarding the randomization and concealment process were the primary contributors to bias. The overall and individual study bias assessments are presented in Figures S1 and S2.

### 3.3. Efficacy Analysis

#### 3.3.1. Ulcer Healing Rates

**Figure 2:**
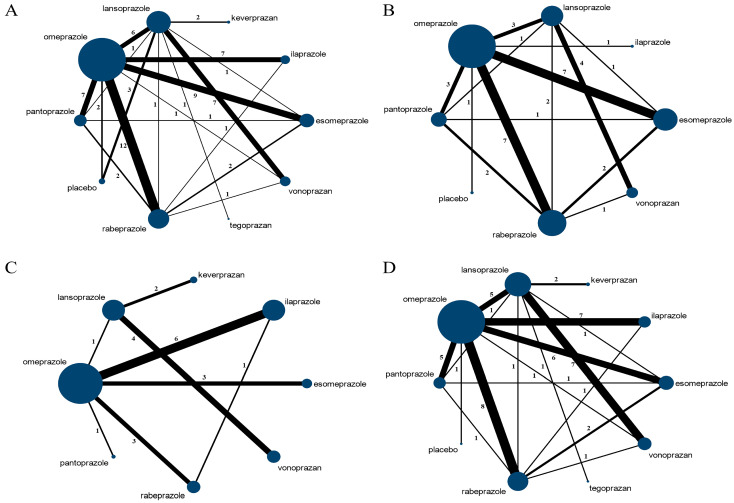
Network map of included RCTs with available direct comparisons for ulcer healing rate, *H. pylori* eradication rate, pain symptom remission rate, and adverse events: (**A**) Ulcer healing rates; (**B**) *H. pylori* eradication rates; (**C**) Pain symptom remission rates; (**D**) Adverse events. The nodes represent different interventions; the node sizes correspond to the number of participants randomly assigned to different therapeutic measures. The width of lines represents the number of studies the direct comparison interventions. The thicker the lines are, the more studies correspond to pairwise direct comparison. RCTs: randomized controlled trials; *H. pylori*: *Helicobacter pylori*.

Ulcer healing rate was evaluated across 56 studies, with a total of 9415 participants, encompassing 10 therapeutic interventions. Figure 2A displays the network map illustrating ulcer healing rates associated with various acid-suppressive drugs used in peptic ulcer treatment. The most common treatment comparisons were omeprazole vs. rabeprazole (12 studies, 1189 participants), followed by omeprazole vs. esomeprazole (9 studies, 825 participants).

**Figure 3:**
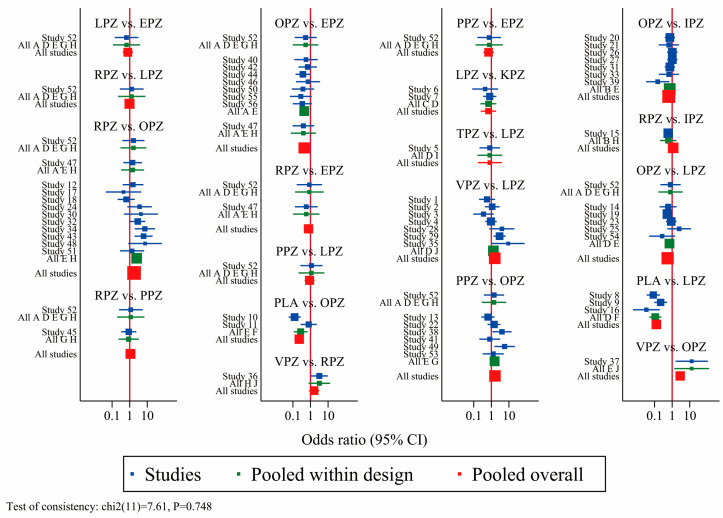
Network forest plot for all direct and mixed comparisons of ulcer healing rates. VPZ: vonoprazan; TPZ: tegoprazan; KPZ: keverprazan; OPZ: omeprazole; PPZ: pantoprazole; LPZ: lansoprazole; RPZ: rabeprazole; EPZ: esomeprazole; IPZ: ilaprazole; PLA: placebo. The pooled effect of a treatment in the comparison set (also called “pooled within design”) and the pooled overall effect (also called “pooled overall”) are marked in green and red, respectively. The blue squares in the center of the lines represent the estimate of the effect of a single study, and the blue (green/red) lines represent its confidence intervals.

**Figure 4:**
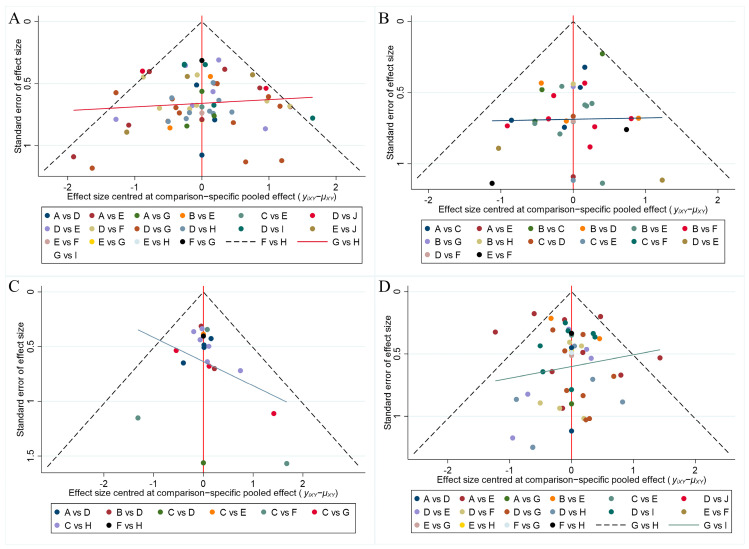
A funnel plot of publication bias in research reports: (**A**) Ulcer healing rates; (**B**) *H. pylori* eradication rates; (**C**) Pain symptom remission rates; (**D**) Adverse events. *H. pylori*: *Helicobacter pylori.*

The pooled ulcer healing rates in the vonoprazan, keverprazan and tegorazan groups were 95.5%, 95.0%, and 94.8%. The ulcer healing rate of each P-CAB was higher than that of each PPI (Table S7). The network forest plot of Figure 3 shows the ORs (95% CI) of all 67 individual direct pair comparisons on 19 regimen pairwise meta-analyses. Of these, the comparisons of vonoprazan vs. omeprazole (OR: 2.84; 95% CI: 1.52–5.30), omeprazole vs. esomeprazole (OR: 0.43; 95% CI: 0.26–0.72), placebo vs. omeprazole (OR: 0.23; 95% CI: 0.12–0.43), and placebo vs. lansoprazole (OR: 0.13; 95% CI: 0.07–0.23) yielded significant results. In the network meta-analysis, no significant inconsistency was identified for ulcer healing rates analysis by the global inconsistency test (Figure 3) and the local inconsistency test (Figure S3), thereby satisfying the assumption for using the consistency model. The corresponding funnel plot of Figure 4A appears symmetrical, implying the absence of publication bias or small study effects.

**Figure 5:**
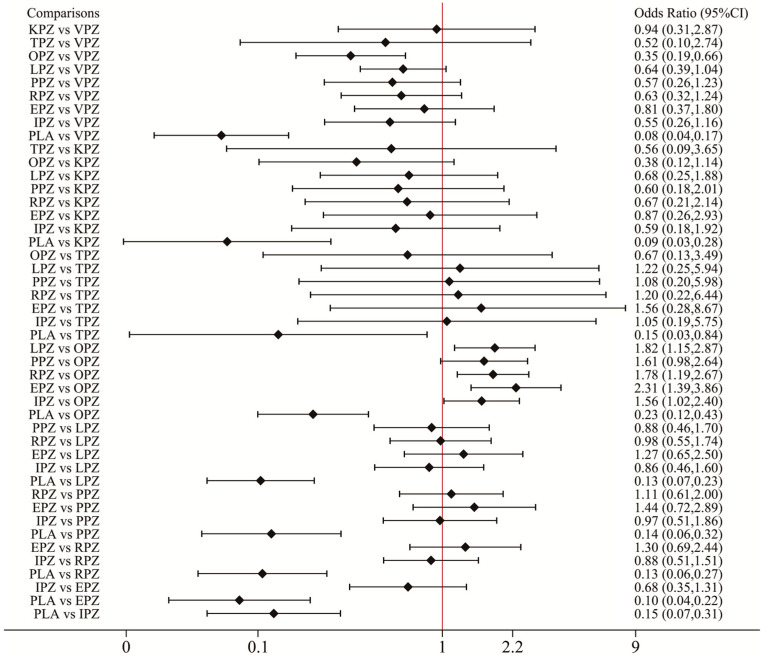
Forest plot of network meta-analysis results for ulcer healing rate. Network forest plots (OR, 95% CI) showed the result of direct and indirect comparisons between interventions. OR: odds ratio; 95% CI: 95% confidence intervals; VPZ: vonoprazan; TPZ: tegoprazan; KPZ: keverprazan; OPZ: omeprazole; PPZ: pantoprazole; LPZ: lansoprazole; RPZ: rabeprazole; EPZ: esomeprazole; IPZ: ilaprazole; PLA: placebo.

**Figure 6:**
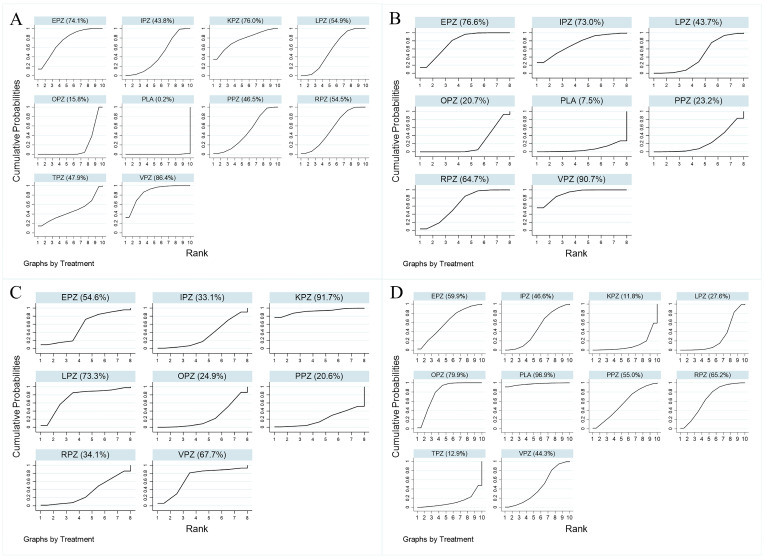
The cumulative rank probability plot of ulcer healing rate, *H. pylori* eradication rate, pain symptom remission rate and adverse events under different treatment regimens: (**A**) Ulcer healing rates; (**B**) *H. pylori* eradication rates; (**C**) Pain symptom remission rates; (**D**) Adverse events. VPZ: vonoprazan; TPZ: tegoprazan; KPZ: keverprazan; OPZ: omeprazole; PPZ: pantoprazole; LPZ: lansoprazole; RPZ: rabeprazole; EPZ: esomeprazole; IPZ: ilaprazole; PLA: placebo; *H. pylori*: *Helicobacter pylori.*

The network forest plot of Figure 5 shows the ORs (95% CI) of all 45 direct and indirect comparisons in this network meta-analysis (19 direct and 26 indirect). Significant differences were observed in ulcer healing rates between all acid-suppressive drugs and placebo. In addition, the ulcer healing rates of vonoprazan, lansoprazole, rabeprazole, esomeprazole, and ilaprazole were significantly superior to those of omeprazole. No significant difference in healing rate was observed between other PPIs and P-CABs for the treatment of peptic ulcers. In Figure 6A, the results of rankograms and SUCRA values indicated that vonoprazan (86.4%) had the best healing effect, followed by keverprazan (76.0%), esomeprazole (74.1%), lansoprazole (54.9%), rabeprazole (54.5%), tegoprazan (47.9%), pantoprazole (46.5%), ilaprazole (43.8%), omeprazole (15.8%), and placebo (0.2%). 

#### 3.3.2. *Helicobacter pylori* Eradication Rates

A total of 24 studies (3250 participants) involving eight therapeutic measures reported *H. pylori* eradication rates. The network map (Figure 2B) presents *H. pylori* eradication rates associated with different acid-suppressive drugs in the treatment of peptic ulcers combined with *H. pylori* infection. The most frequently studied comparisons were omeprazole vs. esomeprazole (7 studies, 689 participants) and omeprazole vs. rabeprazole (7 studies, 614 participants).

The pooled *H. pylori* eradication rates for each intervention are summarized in Table S8, and the results show that the eradication rates of vonoprazan are the highest (92.9%). The network forest plot (Figure S4) displays the ORs (95% CI) for all 35 individual direct pair comparisons across 14 regimen pairwise meta-analyses. Significant findings were observed for omeprazole vs. esomeprazole (OR: 0.46; 95% CI: 0.29–0.71), pantoprazole vs. esomeprazole (OR: 0.42; 95% CI: 0.22–0.93), rabeprazole vs. omeprazole (OR: 1.83; 95% CI: 1.18–2.85), and vonoprazan vs. lansoprazole (OR: 2.09; 95% CI: 1.28–3.40). No significant inconsistency was identified in the network meta-analysis, as confirmed by the global inconsistency test (Figure S4) and the local inconsistency test (Figure S5), thereby supporting the utilization of the consistency model. The funnel plot (Figure 4B) suggests a symmetrical distribution, indicating the absence of publication bias or small study effects.

**Figure 7:**
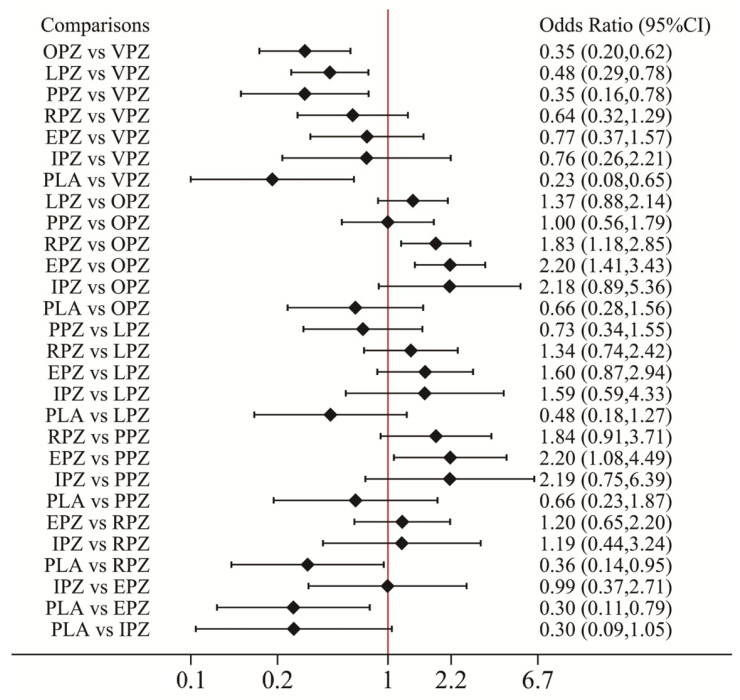
Forest plot of network meta-analysis results for *H. pylori* eradication rate. Network forest plot (OR, 95% CI) showed the result of direct and indirect comparisons between interventions. *H. pylori*: *Helicobacter pylori*; OR: odds ratio; 95% CI: 95% confidence intervals; VPZ: vonoprazan; TPZ: tegoprazan; KPZ: keverprazan; OPZ: omeprazole; PPZ: pantoprazole; LPZ: lansoprazole; RPZ: rabeprazole; EPZ: esomeprazole; IPZ: ilaprazole; PLA: placebo.

The network forest plot (Figure 7) summarizes the ORs (95% CI) for all 28 direct and indirect comparisons in this network meta-analysis (14 direct and 14 indirect). Vonoprazan demonstrated significantly superior eradication rates compared to omeprazole, lansoprazole, pantoprazole, and placebo. However, there are no significant differences in the eradication rates between vonoprazan and rabeprazole, esomeprazole, and ilaprazole for the treatment of peptic ulcers combined with *H. pylori* infection. The results of rankograms and SUCRA values (Figure 6B) indicate that vonoprazan (90.7%) exhibited the most effective eradication, followed by esomeprazole (76.6%), ilaprazole (73.0%), rabeprazole (64.7%), lansoprazole (43.7%), pantoprazole (23.2%), omeprazole (20.7%), and placebo (7.5%).

#### 3.3.3. Remission Rate of Ulcer-Related Symptoms

A total of 21 studies reported remission rates of ulcer-related symptoms, with twenty-one studies containing eight interventions analyzing pain remission rates (Figure 2C), ten studies containing six interventions analyzing abdominal distension remission rates (Figure S6), twelve studies containing six interventions analyzing nausea and vomiting remission rates (Figure S7), six studies containing five interventions analyzing heartburn remission rates (Figure S8), five studies with five interventions analyzed regurgitation remission rates (Figure S9), four studies with four interventions analyzed epigastric burning sensation remission rates (Figure S10), four studies with two interventions analyzed lack of appetite remission rates (Figure S11), and two studies with two interventions analyzed belching remission rates (Figure S12).

**Figure 8:**
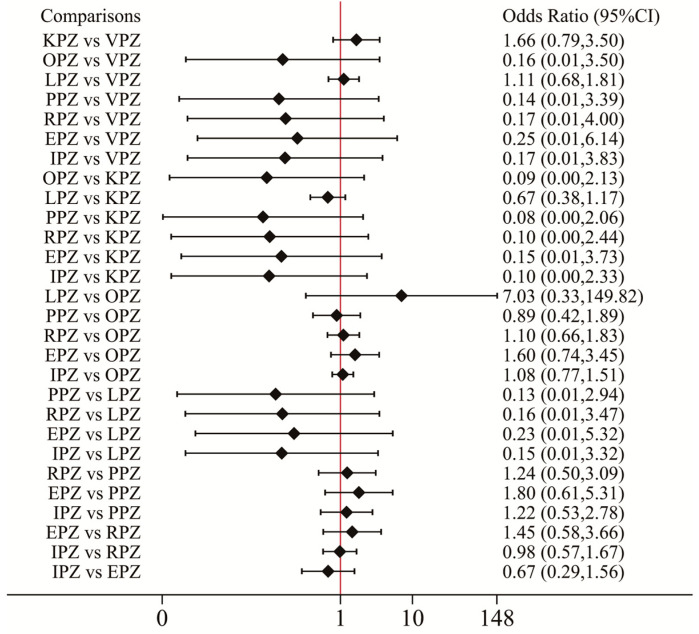
Forest plot of network meta-analysis results for pain symptom remission rate. Network forest plot (OR, 95% confidence intervals) showed the result of direct and indirect comparisons between interventions. OR: odds ratio; 95% CI: 95% confidence intervals; VPZ: vonoprazan; KPZ: keverprazan; OPZ: omeprazole; PPZ: pantoprazole; LPZ: lansoprazole; RPZ: rabeprazole; EPZ: esomeprazole; IPZ: ilaprazole.

The absence of significant inconsistency was confirmed by the global inconsistency test (Figure S13) and the local inconsistency test (Figure S14); thus, the consistency model was used to assess the pain remission rates for different interventions. Despite the SUCRA results (Figure 6C) showing that keverprazan (91.7%) exhibited the highest pain remission rate, followed by lansoprazole (73.3%), vonoprazan (67.7%), esomeprazole (54.6%), pairwise comparisons revealed no significant difference in pain remission rates among the different interventions (Figure 8).

Since only the interventions for pain remission rates formed a loop, whereas the other outcome measures did not establish network connections, we conducted a pair-to-group analysis using conventional meta-analysis. Overall, ilaprazole demonstrated superior efficacy compared to omeprazole in alleviating abdominal distension symptoms. Vonoprazan exhibited better efficacy in alleviating heartburn symptoms compared to lansoprazole.

### 3.4. Safety Analysis

**Figure 9:**
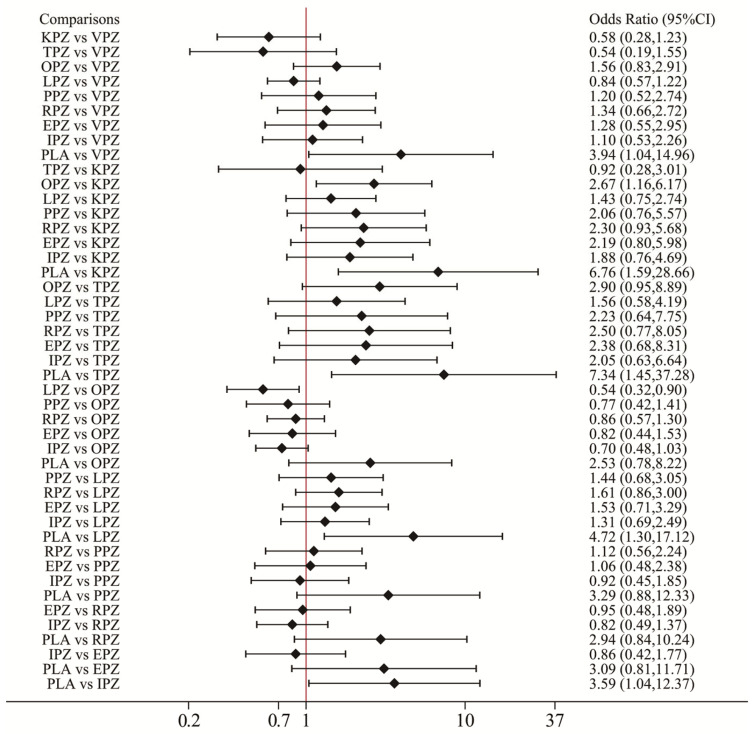
Forest plot of network meta-analysis results for adverse events. Network forest plot (OR, 95% CI) showed the result of direct and indirect comparisons between interventions. OR: odds ratio; 95% CI: 95% confidence intervals; VPZ: vonoprazan; TPZ: tegoprazan; KPZ: keverprazan; OPZ: omeprazole; PPZ: pantoprazole; LPZ: lansoprazole; RPZ: rabeprazole; EPZ: esomeprazole; IPZ: ilaprazole; PLA: placebo.

The adverse events were reported in 41 studies (8168 participants) involving 10 different therapeutic measures. Figure 2D shows the network map of adverse events. No significant inconsistency was identified for safety analysis by the global inconsistency test (Figure S15) and the local inconsistency test (Figure S16). The corresponding funnel plot appears symmetrical in Figure 4D, implying the absence of publication bias or small study effects. As shown in Figure 9, the incidence of adverse events was significantly lower for vonoprazan, keverprazan, tegoprazan, lansoprazole, and ilaprazole compared with placebo. The SUCRA results showed that placebo caused the highest incidence of adverse events (96.9%), followed by omeprazole (79.9%). Keverprazan (11.8%) and tegoprazan (12.9%) had a lower incidence of adverse events (Figure 6D).

Twenty-three studies assessed the safety of P-CABs or PPIs monotherapy, and 18 studies provided safety data for P-CABs or PPIs combination antibiotics as eradication regimens. Figure S17 presents the pairwise comparisons for monotherapy (Figure S17A) and combination therapy (Figure S17B). Compared to the placebo, the incidence of adverse events in the monotherapy group was significantly lower for vonoprazan, keverprazan, lansoprazole, pantoprazole, and ilaprazole. Notably, the incidence of adverse events was significantly higher for omeprazole compared to keverprazan. In the combination antibiotic therapy group, there were no significant differences in safety outcomes among the various P-CABs and PPIs due to the absence of a placebo in the analysis. Figure S18 presents the SUCRA analysis, ranking the safety of treatments based on the incidence of adverse events. These results are generally consistent with the results of the overall safety analysis.

As shown in Figure S19, the meta-analysis of drug-related adverse events revealed significant differences in the comparison of vonoprazan vs. lansoprazole (OR: 2.15; 95% CI: 1.60–2.89) and rabeprazole vs. ilaprazole (OR: 3.71; 95% CI: 1.99–6.91). There was no significant difference in the incidence of drug-related adverse events between other P-CABs and PPIs. As shown in Figure S20, the incidence of serious adverse events was also significantly higher in vonoprazan compared with lansoprazole (OR: 2.22; 95% CI: 1.11–4.42).

### 3.5. Ranking of Efficacy and Safety 

Combining the SUCRA results of ulcer healing rates and *H. pylori* eradication rates, vonoprazan had a better curative effect, followed by esomeprazole (Figure S21A). Combining the SUCRA results of ulcer healing rates and pain symptom remission rates, keverprazan showed good performance in relieving pain and promoting ulcer healing (Figure S21B). Based on the comprehensive synthesis of peptic ulcer healing rates and adverse events SUCRA results, it was observed that vonoprazan demonstrated the most favorable therapeutic effects in the treatment of peptic ulcers. However, adverse events showed a moderate risk. Vonoprazan has a higher risk of adverse events compared to lansoprazole, tegoprazan, and keverprazan. Keverprazan exhibited relatively good efficacy in treating peptic ulcers, with a lower incidence of adverse events. Conversely, placebo and omeprazole showed poorer therapeutic efficacy and a higher occurrence of adverse events (Figure S21C).

### 3.6. Subgroup Analysis 

#### 3.6.1. Regional Effect

Since P-CABs have not been used in regions other than Asia, they were divided into the following three geographic regions based on where the RCTs were conducted: Asia, Europe, and North America. Table 1 shows the mean healing rates for the relevant regions, pairwise comparisons compared with placebo, SUCRA values for each regimen, and overall data. In Asia, the best ulcer healing results were seen with vonoprazan (88.0%) and keverprazan (81.5%). In both Europe and North America, esomeprazole (75.5% and 78.6%) had the best performance.

#### 3.6.2. Ulcer Location Effect

Based on the location of ulcer occurrence, we performed a subgroup analysis of duodenal ulcers and gastric ulcers. Thirty-four studies reported the results of duodenal ulcer healing rates, while 16 studies reported the results of gastric ulcer healing rates. The results of SUCRA in both groups consistently showed the best treatment effect of vonoprazan. Other interventions were significantly better than placebo in pairwise comparisons (Table 1).

#### 3.6.3. *Helicobacter pylori* Infection Effect

To investigate the effect of *H. pylori* infection, we performed a subgroup analysis, dividing the RCTs into two groups. Thirty-four studies reported ulcer healing rates in *H. pylori*-positive patients, and ten studies reported ulcer healing rates in *H. pylori*-negative patients. Table 1 shows the results of the *H. pylori*-positive subgroup. Compared to placebo, both P-CABs and PPIs yielded significant results. The SUCRA results demonstrated that the healing rate of vonoprazan (94.0%) was the highest, followed by keverprazan (69.0%). Because the eight interventions involved in the *H. pylori*-negative peptic ulcer study did not form a network, we performed a general meta-analysis. As shown in Figure S22, the pooled results showed that there was no statistically significant difference in ulcer healing rates in *H. pylori*-negative patients among the different treatment interventions in pairwise comparisons.

#### 3.6.4. Treatment Duration Effect

Generally, duodenal ulcers are treated for 6 weeks, and gastric ulcers are treated for 8 weeks. We categorized the treatment duration into three groups: less than 4 weeks, 4–6 weeks, and greater than 6 weeks (Table 1). In the group with a treatment duration of less than 4 weeks, the results showed that vonoprazan had the highest ulcer healing rate and SUCRA value compared to other treatment regimens. In the 4–6 weeks treatment group, vonoprazan (96.5%) had the highest ulcer healing rate. However, the SUCRA results indicated that esomeprazole and keverprazan exhibited better efficacy. In the group with a treatment duration greater than 6 weeks, pairwise comparisons revealed no significant differences (Figure S23).

## 4. Discussion

In this systematic review and network meta-analysis, we comprehensively compared the efficacy and safety of P-CABs versus PPIs in the treatment of peptic ulcers with or without *H. pylori* infection. The results showed that vonoprazan was significantly superior to omeprazole and placebo in ulcer healing rates, while no significant difference was found between vonoprazan and other PPIs. In terms of the *H. pylori* eradication rate, vonoprazan was significantly better than omeprazole, lansoprazole, pantoprazole, and the placebo, while there was no significant difference with other PPIs. In terms of ulcer-related symptom remission rates, vonoprazan was significantly better than lansoprazole in relieving heartburn symptoms. Combining the SUCRA ranking results for ulcer healing rates and *H. pylori* eradication rates, vonoprazan has superior efficacy in the healing of peptic ulcers, underscoring its potential as a promising alternative to traditional PPIs.

Keverprazan also demonstrated promising efficacy in ulcer healing rates and pain remission rates. Keverprazan was approved in China in February 2023 [16]. Based on the structure of vonoprazan, keverprazan changes the pyridine into a benzene ring with an ether chain, altering its lipid solubility, enhancing its water solubility, and thereby changing its tissue distribution, particularly increasing its distribution in the target organ [78]. Consequently, it also achieves a good acid inhibition effect. A clinical study reported that after a single dose of 20 mg keverprazan, the percentage of time with gastric pH >3, >4, and >5 exceeded 80% for 24 h, and during the night, the percentage of time with gastric pH >3, >4, and >5 exceeded 95% [79].

With the increasing antibiotic resistance to *H. pylori* and the widespread use of NSAIDs, the treatment of peptic ulcers has become increasingly difficult [80,81]. Several factors contribute to the failure of PPI treatment, including the following: PPIs are precursor drugs that need to be activated in an acidic environment to be effective, so the onset of action is slow; they are unstable in an acidic environment and need to be made into enteric-soluble preparations. They are easily affected by food, limiting the timing of medication intake; they are susceptible to the polymorphism of the CYP2C19 gene, and the extensive metabolizer of CYP2C19 cannot achieve a better acid inhibition effect [15,82]. Vonoprazan has a fast effect and can rapidly increase intragastric pH, putting *H. pylori* in a replicating state, thus enhancing the antimicrobial sensitivity of antibiotics and further strengthening the bactericidal effect of unstable antibiotics in an acidic environment. This helps clarithromycin and amoxicillin function in an ideal pH environment, promoting the healing of ulcers and eradication of *H. pylori* [83,84,85]. The results of this study showed that vonoprazan not only showed a good effect on ulcer healing rates but also in eradicating *H. pylori*, which could fundamentally solve the problem of peptic ulcers. *H. pylori* infection is the main factor that causes gastric acid and pepsin damage to gastric mucosa and is also one of the main reasons for peptic ulcer and its easy recurrence [6]. Eradication of *H. pylori* can promote mucosal ulcer wound healing and reduce the recurrence of peptic ulcer. The 2020 Japan Society of Gastroenterology Evidence-Based Clinical Practice Guidelines for Peptic Ulceration recommends vonoprazan-based triple regimens as the first-line regimen for *H. pylori* eradication [86]. The Maastricht VI/Florence Consensus report also recommended vonoprazan combined with antibiotics as first- and second-line treatment, especially in patients with evidence of antibiotic-resistant infections [87]. Different studies have drawn various conclusions about the mechanism of peptic ulcers induced by *H. pylori* infection: they can change the bacterial community function and microbial species in the digestive tract, induce virulence factors such as blood group antigen adhesion, outer inflammatory protein adhesion, urease, and vacuolar cytotoxin, and increase the secretion of gastric acid, thus damaging the digestive mucosa and inducing peptic ulcer [1,88,89].

However, the SUCRA results of this study showed that vonoprazan had a moderate risk of adverse events, and the risk of drug-related adverse events and serious adverse events was higher than that of lansoprazole. This finding is consistent with the results of a previous meta-analysis on peptic ulcers. This meta-analysis found that P-CAB treatment was associated with an increased risk of serious adverse events compared to lansoprazole [18]. Additionally, a meta-analysis on acid-related diseases also found that the incidence of adverse events in patients with duodenal ulcers treated with vonoprazan was significantly higher than that with PPIs [90]. Another study evaluated the safety of vonoprazan in various indications, and the results showed that patients with peptic ulcers had a higher rate of adverse events than other acid-related diseases such as gastroesophageal reflux and *H. pylori* infection [91]. This may be due to the fact that peptic ulcers are more complex and require longer treatment than other diseases. The most common adverse events associated with vonoprazan include increased serum gastrin levels, followed by increased pepsinogen I levels, nasopharyngitis, bloating, loose stools, and so on [91,92]. In addition, studies have reported that vonoprazan can affect human gut microbiota [93]. This study showed that keverprazan had a lower incidence of adverse events compared to vonoprazan. Its favorable safety profile suggests that keverprazan may be a preferred choice for certain patient populations, especially those who are intolerant or have an increased risk of adverse drug reactions.

In our network meta-analysis, there may be an overstatement of the therapeutic effect of P-CABs and an underestimation of the therapeutic effect of PPIs. Therefore, we performed subgroup analyses to explore the effects of *H. pylori* infection, ulcer location, treatment duration, and regional differences on ulcer healing rates. In patients with *H. pylori*-positive peptic ulcer, vonoprazan was at the top of the SUCRA rankings for ulcer healing rates, followed by keverprazan. In patients with *H. pylori*-negative peptic ulcer, pairwise comparisons showed no significant difference between each P-CAB and PPI. This may be due to the fact that *H. pylori*-positive peptic ulcer patients require a more acid-suppressive environment. Studies have shown that different acid-related diseases require specific intragastric pH levels [7]. Treatment of peptic ulcers should raise intragastric pH >3 for more than 18 h a day [94]. Treatment of gastroesophageal reflux requires maintaining a pH >4 for more than 18 h a day [95]. For the eradication of *H. pylori*, the intragastric pH is required to be >5 for more than 18 h a day. The acid suppression criteria for peptic ulcers were the lowest, which may be the reason why there was no significant difference between P-CAB and PPI in the treatment of patients with *H. pylori*-negative peptic ulcer. By analyzing the different ulcer locations, it was found that vonoprazan was more effective in the treatment of duodenal ulcers and gastric ulcers. By analyzing subgroups of treatment duration, it was found that the SUCRA results of vonoprazan were significantly higher than those of PPIs for treatment durations of less than 4 weeks. This further illustrates the rapid onset of P-CAB, which does not require acid and proton pump activation to achieve the desired effect [96]. However, the acid-suppressive effect of PPIs was gradually appeared over time. As RCTs of P-CABs have been conducted mainly in Asia, it is not possible to compare the efficacy of P-CABs for peptic ulcer treatment in other regions at this time. In Asia, both vonoprazan and keverprazan have shown better therapeutic efficacy. Future studies will need to delve deeper into the efficacy of P-CABs in other regions.

There are some limitations in this study. First, our study did not explore the use of combined NSAIDs. However, it is important to note that, based on the baseline of the included studies, most of the studies explicitly stated the exclusion of patients using NSAIDs in the exclusion criteria, which avoided the influence of NSAIDs on the efficacy of acid-suppressive drugs in the treatment of peptic ulcer. Second, CYP2C19 genotype is a key factor influencing the acid suppression effect of PPIs, but the limited number of studies reporting genotype results precluded a comprehensive meta-analysis to assess its impact on treatment efficacy [54]. Third, there are several potential confounders in this study. For example, alcohol consumption, tobacco use, fasting, and cancer treatment with angiogenesis inhibitors are also risk factors for the development of gastric and duodenal ulcers, but these factors were not adjusted for in the original studies [97]. In addition, only one RCT of tegoprazan met the inclusion criteria, and there were only two studies on keverprazan, so the combined efficacy of these two P-CABs should be interpreted with caution. More studies are needed to further characterize the efficacy and safety of P-CABs other than vonoprazan.

## 5. Conclusions

In conclusion, vonoprazan showed superior performance in the ulcer healing rate and *H. pylori* eradication rate, especially for patients with *H. pylori*-positive peptic ulcers. Keverprazan has also shown good therapeutic outcomes and has performed better in terms of safety. However, the association of P-CABs with adverse events should be noted. Overall, P-CABs show potential as a new class of drugs in the treatment of peptic ulcers and may be an alternative to traditional PPIs in specific cases. Further studies are needed to clarify the long-term efficacy and safety of P-CABs in clinical practice to guide clinical decisions and provide better treatment options.

## Figures and Tables

**Table 1:** Overall and subgroup data, including ulcer healing rates, pairwise comparisons, and the surface under the cumulative ranking values across different continents, ulcer locations, *H. pylori* infection status, and treatment duration in this network meta-analysis.

Variable	Overall Data	Continent			Ulcer Location Effect	
		Asia	Europe	North America	Duodenal Ulcer	Gastric Ulcer
Ulcer healing rates, % (95% CI)						
Regimen						
vonoprazan	95.5 (94.2–96.7)	95.5 (94.2–96.7)	—	—	95.8 (94.3–97.3)	94.1 (91.4–96.8)
keverprazan	95.0 (92.3–97.8)	95.0 (92.3–97.8)	—	—	95.0 (92.3–97.8)	—
tegoprazan	94.8 (90.3–99.2)	94.8 (90.3–99.2)	—	—	—	94.8 (90.3–99.2)
omeprazole	84.8 (82.3–87.3)	83.3 (80.3–86.3)	90.4 (86.3–94.5)	77.8 (70.9–84.8)	85.5 (82.9–88.1)	81.7 (77.3–86.2)
lansoprazole	91.7 (89.5–93.9)	92.9 (90.5–95.3)	90.5 (86.6–94.5)	85.2 (76.4–94.1)	90.9 (88.0–93.8)	90.8 (87.3–94.3)
pantoprazole	90.9 (88.5–93.2)	91.1 (87.9–94.3)	88.5 (79.9–97.1)	—	90.9 (88.1–93.8)	90.6 (86.9–94.4)
rabeprazole	89.3 (85.2–93.3)	87.7 (82.4–93.0)	97.5 (94.9–100.0)	88.9 (78.6–99.2)	89.7 (84.7–94.7)	87.6 (82.2–92.9)
esomeprazole	92.1 (89.5–94.7)	92.1 (89.5–94.7)	—	—	91.0 (87.7–94.2)	93.2 (89.4–97.0)
ilaprazole	88.7 (84.0–93.4)	88.7 (84.0–93.4)	—	—	91.3 (87.5–95.1)	63.9 (52.8–75.0)
placebo	45.0 (18.1–72.0)	20.0 (0–40.2)	86.6 (78.4–94.7)	38.2 (26.8–49.6)	45.0 (18.1–72.0)	20.0 (0–40.2)
Pairwise comparisons, OR (95% CI)						
Comparison	placebo	placebo	placebo	placebo	placebo	placebo
vonoprazan	12.38 (5.76–26.60)	25.95 (3.30–204.25)	—	—	16.32 (6.86–38.80)	54.56 (7.24–411.37)
keverprazan	11.59 (3.56–37.73)	17.20 (2.22–133.27)	—	—	11.86 (3.50–40.12)	—
tegoprazan	6.48 (1.19–35.23)	32.03 (5.05–349.76)	—	—	—	23.78 (1.96–289.05)
omeprazole	4.36 (2.32–8.18)	28.97 (4.39–191.38)	1.26 (0.34–4.69)	7.97 (2.16–29.36)	4.27 (2.21–8.25)	11.93 (1.48–96.08)
lansoprazole	7.92 (4.30–14.57)	10.93 (1.46–81.84)	1.53 (0.34–6.86)	7.27 (2.80–18.85)	8.06 (4.27–15.23)	29.07 (4.21–200.80)
pantoprazole	7.00 (3.17–15.46)	23.65 (2.96–188.96)	1.22 (0.26–5.73)	—	5.41 (2.11–13.85)	29.26 (3.38–253.24)
rabeprazole	7.75 (3.72–16.14)	20.90 (2.74–159.18)	2.23 (0.32–15.72)	12.34 (1.43–106.22)	9.60 (4.23–21.79)	22.29 (2.71–183.34)
esomeprazole	10.07 (4.50–22.53)	23.70 (2.09–269.02)	—	—	9.68 (3.97–23.61)	29.76 (3.29–269.28)
ilaprazole	6.81 (3.19–14.55)	42.62 (6.09–298.36)	—	—	7.63 (3.37–17.27)	11.73 (1.12–122.69)
SUCRA, % Regimen						
vonoprazan	86.4	88.0	—	—	92.7	94.1
keverprazan	76.0	81.5	—	—	73.6	—
tegoprazan	47.9	54.0	—	—	—	55.0
omeprazole	15.8	13.5	42.8	61.6	16.3	22.1
lansoprazole	54.9	65.2	58.7	59.5	53.1	65.0
pantoprazole	46.5	55.1	41.4	—	31.1	66.9
rabeprazole	54.5	46.6	75.5	78.6	66.9	51.5
esomeprazole	74.1	61.9	—	—	66.4	68.1
ilaprazole	43.8	33.9	—	—	49.9	27.0
placebo	0.2	0.1	31.6	0.4	0.0	0.4
Variable	Overall Data	*H. pylori* Infection Status		Treatment Duration		
		*H. pylori* Negative	*H. pylori* Positive	<4 Weeks	4–6 Weeks	>6 Weeks
Ulcer healing rates, % (95% CI)						
Regimen						
vonoprazan	95.5 (94.2–96.7)	90.3 (83.1–97.6)	95.6 (94.0–97.2)	94.4 (88.1–100.0)	96.5 (94.9–98.2)	92.5 (90.0–94.9)
keverprazan	95.0 (92.3–97.8)	96.6 (89.9–100.0)	83.4 (77.5–89.4)	—	95.0 (92.3–97.8)	—
tegoprazan	94.8 (90.3–99.2)	100	100	—	—	94.8 (90.3–99.2)
omeprazole	84.8 (82.3–87.3)	81.5 (70.8–92.1)	82.3 (78.7–85.9)	77.4 (69.0–85.8)	86.3 (83.7–88.8)	85.7 (79.5–91.8)
lansoprazole	91.7 (89.5–93.9)	86.3 (78.0–94.7)	88.4 (83.5–93.2)	88.0 (82.6–93.4)	92.3 (89.7–94.9)	91.4 (86.0–96.8)
pantoprazole	90.9 (88.5–93.2)	—	87.6 (83.3–91.9)	92.7 (85.9–99.6)	90.6 (88.1–93.1)	78.6 (69.8–87.3)
rabeprazole	89.3 (85.2–93.3)	86.4 (72.0–100.7)	90.2 (86.5–93.8)	87.3 (79.6–95.0)	90.2 (85.0–95.3)	88.9 (78.6–99.2)
esomeprazole	92.1 (89.5–94.7)	—	92.2 (89.4–95.0)	92.0 (88.4–95.6)	92.1 (88.4–95.9)	—
ilaprazole	88.7 (84.0–93.4)	80.0 (66.2–93.8)	91.6 (87.2–96.0)	—	88.7 (84.0–93.4)	—
placebo	45.0 (18.1–72.0)	90.2 (82.0–98.4)	46.2 (31.6–60.9)	—	45.0 (18.1–72.0)	—
Pairwise comparisons, OR (95% CI)						
Comparison	placebo	—	placebo	omeprazole	placebo	—
vonoprazan	12.38 (5.76–26.60)	—	24.03 (5.54–104.31)	10.77 (3.80–30.56)	7.97 (2.89–22.02)	—
keverprazan	11.59 (3.56–37.73)	—	13.23 (2.60–67.35)	—	11.16 (3.73–33.45)	—
tegoprazan	6.48 (1.19–35.23)	—	10.25 (0.15–696.96)	—	—	—
omeprazole	4.36 (2.32–8.18)	—	4.57 (1.13–18.53)	—	4.48 (2.48–8.07)	—
lansoprazole	7.92 (4.30–14.57)	—	11.22 (2.80–44.96)	1.76 (0.63–4.88)	7.73 (4.38–13.64)	—
pantoprazole	7.00 (3.17–15.46)	—	7.81 (1.72–35.53)	1.85 (0.56–6.07)	6.69 (3.10–14.44)	—
rabeprazole	7.75 (3.72–16.14)	—	11.01 (2.58–46.93)	3.70 (2.07–6.60)	5.69 (2.61–12.41)	—
esomeprazole	10.07 (4.50–22.53)	—	11.37 (2.57–50.38)	2.15 (1.17–3.93)	11.33 (4.58–28.01)	—
ilaprazole	6.81 (3.19–14.55)	—	7.08 (1.61–31.19)	—	6.53 (3.22–13.24)	—
SUCRA, % Regimen						
vonoprazan	86.4	—	94.0	99.5	63.3	—
keverprazan	76.0	—	69.0	—	80.2	—
tegoprazan	47.9	—	54.4	—	—	—
omeprazole	15.8	—	16.0	5.7	17.9	—
lansoprazole	54.9	—	61.9	36.1	63.0	—
pantoprazole	46.5	—	41.0	38.1	51.8	—
rabeprazole	54.5	—	62.0	74.4	39.0	—
esomeprazole	74.1	—	63.9	46.2	84.7	—
ilaprazole	43.8	—	35.9	—	50.2	—
placebo	0.2	—	1.9	—	0.0	—

## Data Availability

Data are contained within the article and Supplementary Materials.

## Supplementary Materials

The following supporting information can be downloaded at https://www.mdpi.com/article/10.3390/ph17060698/s1, Table S1. PRISMA checklist; Table S2. Search strategy; Table S3. The basic characteristics of included studies on duodenal ulcer (N = 28); Table S4. The basic characteristics of included studies on gastric ulcer (N = 10); Table S5. The basic characteristics of included studies on duodenal ulcer and gastric ulcer (N = 6); Table S6. The basic characteristics of included studies on peptic ulcer with unspecified location (N = 12); Table S7. The overall ulcer healing rates of P-CABs and PPIs in the meta-analysis; Table S8. The overall *H. pylori* eradication rates of P-CABs and PPIs in the meta-analysis; Figure S1. Risk of bias for included studies assessed by Cochrane risk of bias tool (ROB 2.0); Figure S2. The risk of bias map depicting the proportion of each bias risk item in all studies; Figure S3. Detection of local inconsistencies in ulcer healing rates using node-splitting method; Figure S4. Network forest plot for all direct and mixed comparisons of *H. pylori* eradication rates; Figure S5. Detection of local inconsistencies in *H. pylori* eradication rates using node-splitting method; Figure S6. Forest plot of meta-analysis results for abdominal distension remission rates; Figure S7. Forest plot of meta-analysis results for nausea and vomiting remission rates; Figure S8. Forest plot of meta-analysis results for heartburn remission rates; Figure S9. Forest plot of meta-analysis results for regurgitation remission rates; Figure S10. Forest plot of meta-analysis results for epigastric burning sensation remission rates; Figure S11. Forest plot of meta-analysis results for lack of appetite remission rates; Figure S12. Forest plot of meta-analysis results for belching remission rates; Figure S13. Network forest plot for all direct and mixed comparisons of pain symptom remission rates; Figure S14. Detection of local inconsistencies in pain symptom remission rates using node-splitting method; Figure S15. Network forest plot for all direct and mixed comparisons of adverse events; Figure S16. Detection of local inconsistencies in adverse events using node-splitting method; Figure S17. Forest plot of network meta-analysis results of adverse events for P-CABs and PPIs monotherapy or combination antibiotic therapy. (A) P-CABs and PPIs monotherapy; (B) P-CABs and PPIs combination antibiotic therapy; Figure S18. The cumulative rank probability plot of adverse events for P-CABs and PPIs monotherapy or combination antibiotic therapy. (A) P-CAB and PPIs monotherapy; (B) P-CABs and PPIs combination antibiotic therapy; Figure S19. Forest plot of meta-analysis results for drug-related adverse events; Figure S20. Forest plot of meta-analysis results for serious adverse events; Figure S21. The results of SUCRA for different treatment regimens from the network meta-analysis. (A) SUCRA results of ulcer healing rates and *H. pylori* eradication rates; (B) SUCRA results of ulcer healing rates and pain symptom remission rates; (C) SUCRA results of ulcer healing rates and adverse events; Figure S22. Forest plot of meta-analysis results for ulcer healing rate according to *Helicobacter pylori*-negative peptic ulcer patients; Figure S23. Forest plot of meta-analysis results for 8-week ulcer healing rate.
