# Supplementary material for: Comparative Efficacy and Safety of Potassium-Competitive Acid Blockers vs. Proton Pump Inhibitors for Peptic Ulcer with or without Helicobacter pylori Infection: A Systematic Review and Network Meta-Analysis
Source: Pharmaceuticals (Basel). 2024 May 28;17(6):698. doi: 10.3390/ph17060698 (PMC11206580; doi:10.3390/ph17060698)
Supplement: Supplementary file 1 [file pharmaceuticals-17-00698-s001.zip › pharmaceuticals-3017182-supplementary.pdf]

Supplementary Material

**Supplementary material**	**Page**
**Table S1.** PRISMA checklist	1
**Table S2.** Search strategy	4
**Table S3.** The basic characteristics of included studies on duodenal ulcer (N=28).	9
**Table S4.** The basic characteristics of included studies on gastric ulcer (N=10).	11
**Table S5.** The basic characteristics of included studies on duodenal ulcer and gastric ulcer (N=6).	12
**Table S6.** The basic characteristics of included studies on peptic ulcer with unspecified location (N=12).	13
**Table S7.** The overall ulcer healing rates of P-CABs and PPIs in the meta-analysis.	14
**Table S8.** The overall *H. pylori* eradication rates of P-CABs and PPIs in the meta-analysis.	14
**Figure S1.** Risk of bias for included studies assessed by Cochrane risk of bias tool (ROB 2.0).	15
**Figure S2.** The risk of bias map depicting the proportion of each bias risk item in all studies.	16
**Figure S3.** Detection of local inconsistencies in ulcer healing rates using node-splitting method.	17
**Figure S4.** Network forest plot for all direct and mixed comparisons of *H. pylori* eradication rates.	18
**Figure S5.** Detection of local inconsistencies in *H. pylori* eradication rates using node-splitting method.	19
**Figure S6.** Forest plot of meta-analysis results for abdominal distension remission rates.	20
**Figure S7.** Forest plot of meta-analysis results for nausea and vomiting remission rates.	21
**Figure S8.** Forest plot of meta-analysis results for heartburn remission rates.	22
**Figure S9.** Forest plot of meta-analysis results for regurgitation remission rates.	23
**Figure S10.** Forest plot of meta-analysis results for epigastric burning sensation remission rates.	23
**Figure S11.** Forest plot of meta-analysis results for lack of appetite remission rates.	24
**Figure S12.** Forest plot of meta-analysis results for belching remission rates.	24
**Figure S13.** Network forest plot for all direct and mixed comparisons of pain symptom remission rates.	25
**Figure S14.** Detection of local inconsistencies in pain symptom remission rates using node-splitting method.	25
**Figure S15.** Network forest plot for all direct and mixed comparisons of adverse events.	26
**Figure S16.** Detection of local inconsistencies in adverse events using node-splitting method.	27
**Figure S17.** Forest plot of network meta-analysis results of adverse events for P-CABs and PPIs monotherapy or combination antibiotic therapy. (A) P-CABs and PPIs monotherapy; (B) P-CABs and PPIs combination antibiotic therapy.	28
**Figure S18.** The cumulative rank probability plot of adverse events for P-CABs and PPIs monotherapy or combination antibiotic therapy. (A) P-CAB and PPIs monotherapy; (B) P-CABs and PPIs combination antibiotic therapy.	29

**Figure S19.** Forest plot of meta-analysis results for drug-related adverse events.	30
**Figure S20.** Forest plot of meta-analysis results for serious adverse events.	31
**Figure S21.** The results of SUCRA for different treatment regimens from the network meta-analysis. (A) SUCRA results of ulcer healing rates and *H. pylori* eradication rates; (B) SUCRA results of ulcer healing rates and pain symptom remission rates; (C) SUCRA results of ulcer healing rates and adverse events.	32
**Figure S22.** Forest plot of meta-analysis results for ulcer healing rate according to *Helicobacter pylori*-negative peptic ulcer patients.	33
**Figure S23.** Forest plot of meta-analysis results for 8-week ulcer healing rate.	34
**Reference**	35

1 Supplementary Tables

Table S1. PRISMA checklist

Section and Topic	Item #	Checklist item	Location where item is reported
**TITLE**			
Title	1	Identify the report as a systematic review.	Page 1
**ABSTRACT**			
Abstract	2	See the PRISMA 2020 for Abstracts checklist.	Table S1
**INTRODUCTION**			
Rationale	3	Describe the rationale for the review in the context of existing knowledge.	Page 1-2
Objectives	4	Provide an explicit statement of the objective(s) or question(s) the review addresses.	Page 1-2
**METHODS**			
Eligibility criteria	5	Specify the inclusion and exclusion criteria for the review and how studies were grouped for the syntheses.	Page 2
Information sources	6	Specify all databases, registers, websites, organisations, reference lists and other sources searched or consulted to identify studies. Specify the date when each source was last searched or consulted.	Page 2
Search strategy	7	Present the full search strategies for all databases, registers and websites, including any filters and limits used.	Table S2
Selection process	8	Specify the methods used to decide whether a study met the inclusion criteria of the review, including how many reviewers screened each record and each report retrieved, whether they worked independently, and if applicable, details of automation tools used in the process.	Page 2-3
Data collection process	9	Specify the methods used to collect data from reports, including how many reviewers collected data from each report, whether they worked independently, any processes for obtaining or confirming data from study investigators, and if applicable, details of automation tools used in the process.	Page 3
Data items	10a	List and define all outcomes for which data were sought. Specify whether all results that were compatible with each outcome domain in each study were sought (e.g. for all measures, time points, analyses), and if not, the methods used to decide which results to collect.	Page 3
	10b	List and define all other variables for which data were sought (e.g. participant and intervention characteristics, funding sources). Describe any assumptions made about any missing or unclear information.	Page 3
Study risk of bias assessment	11	Specify the methods used to assess risk of bias in the included studies, including details of the tool(s) used, how many reviewers assessed each study and whether they worked independently, and if applicable, details of automation tools used in the process.	Page 3
Effect measures	12	Specify for each outcome the effect measure(s) (e.g. risk ratio, mean difference) used in the synthesis or presentation of results.	Page 3

Section and Topic	Item #	Checklist item	Location where item is reported
Synthesis methods	13a	Describe the processes used to decide which studies were eligible for each synthesis (e.g. tabulating the study intervention characteristics and comparing against the planned groups for each synthesis (item #5)).	Page 3
	13b	Describe any methods required to prepare the data for presentation or synthesis, such as handling of missing summary statistics, or data conversions.	Page 3
	13c	Describe any methods used to tabulate or visually display results of individual studies and syntheses.	Page 3
	13d	Describe any methods used to synthesize results and provide a rationale for the choice(s). If meta-analysis was performed, describe the model(s), method(s) to identify the presence and extent of statistical heterogeneity, and software package(s) used.	Page 3
	13e	Describe any methods used to explore possible causes of heterogeneity among study results (e.g. subgroup analysis, meta-regression).	Page 3
	13f	Describe any sensitivity analyses conducted to assess robustness of the synthesized results.	/
Reporting bias assessment	14	Describe any methods used to assess risk of bias due to missing results in a synthesis (arising from reporting biases).	Page 3
Certainty assessment	15	Describe any methods used to assess certainty (or confidence) in the body of evidence for an outcome.	/
**RESULTS**			
Study selection	16a	Describe the results of the search and selection process, from the number of records identified in the search to the number of studies included in the review, ideally using a flow diagram.	Page 4, Figure 1
	16b	Cite studies that might appear to meet the inclusion criteria, but which were excluded, and explain why they were excluded.	Figure 1
Study characteristics	17	Cite each included study and present its characteristics.	Page 4, Table S3-S6
Risk of bias in studies	18	Present assessments of risk of bias for each included study.	Page 5, Figures S1-S2
Results of individual studies	19	For all outcomes, present, for each study: (a) summary statistics for each group (where appropriate) and (b) an effect estimate and its precision (e.g. confidence/credible interval), ideally using structured tables or plots.	Page 5-14
Results of syntheses	20a	For each synthesis, briefly summarise the characteristics and risk of bias among contributing studies.	Page 5-14
	20b	Present results of all statistical syntheses conducted. If meta-analysis was done, present for each the summary estimate and its precision (e.g. confidence/credible interval) and measures of statistical heterogeneity. If comparing groups, describe the direction of the effect.	Page 5-14
	20c	Present results of all investigations of possible causes of heterogeneity among study results.	Page 5-14

Section and Topic	Item #	Checklist item	Location where item is reported
	20d	Present results of all sensitivity analyses conducted to assess the robustness of the synthesized results.	Page 13-15, Table 1
Reporting biases	21	Present assessments of risk of bias due to missing results (arising from reporting biases) for each synthesis assessed.	/
Certainty of evidence	22	Present assessments of certainty (or confidence) in the body of evidence for each outcome assessed.	Page 5-14
**DISCUSSION**			
Discussion	23a	Provide a general interpretation of the results in the context of other evidence.	Page 14-16
	23b	Discuss any limitations of the evidence included in the review.	Page 14-16
	23c	Discuss any limitations of the review processes used.	Page 14-16
	23d	Discuss implications of the results for practice, policy, and future research.	Page 14-16
**OTHER INFORMATION**			
Registration and protocol	24a	Provide registration information for the review, including register name and registration number, or state that the review was not registered.	Page 2
	24b	Indicate where the review protocol can be accessed, or state that a protocol was not prepared.	Page 2
	24c	Describe and explain any amendments to information provided at registration or in the protocol.	/
Support	25	Describe sources of financial or non-financial support for the review, and the role of the funders or sponsors in the review.	Page 17
Competing interests	26	Declare any competing interests of review authors.	Page 17
Availability of data, code and other materials	27	Report which of the following are publicly available and where they can be found: template data collection forms; data extracted from included studies; data used for all analyses; analytic code; any other materials used in the review.	Page 17

From: Page MJ, McKenzie JE, Bossuyt PM, Boutron I, Hoffmann TC, Mulrow CD, et al. The PRISMA 2020 statement: an updated guideline for reporting systematic reviews. BMJ 2021;372:n71. doi: 10.1136/bmj.n71

Table S2. Search strategy

(1) Search Strategy for PubMed

ID	Search	Results
#1	((((((((((((((((((vonoprazan[Title/Abstract]) OR (tak-438[Title/Abstract])) OR (tak438[Title/Abstract])) OR (tak 438[Title/Abstract])) OR (tegoprazan[Title/Abstract])) OR (CJ-12420[Title/Abstract])) OR (revaprazan[Title/Abstract])) OR (YH-1885[Title/Abstract])) OR (keverprazan[Title/Abstract])) OR (H-008[Title/Abstract])) OR (KFP-H008[Title/Abstract])) OR (soraprazan[Title/Abstract])) OR (BYK61359[Title/Abstract])) OR (BYK 61359[Title/Abstract])) OR (BYK-61359[Title/Abstract])) OR (linaprazan[Title/Abstract])) OR (AZD-0865[Title/Abstract])) OR (potassium competitive acid blocker[Title/Abstract])) OR (potassium-competitive acid blockers[Title/Abstract])) OR (P-CAB[Title/Abstract]))	806
#2	((((((((proton pump inhibitor[Title/Abstract]) OR (PPI[Title/Abstract])) OR (omeprazole[Title/Abstract])) OR (esomeprazole[Title/Abstract])) OR (lansoprazole[Title/Abstract])) OR (rabeprazole[Title/Abstract])) OR (pantoprazole[Title/Abstract])) OR (dexlansoprazole[Title/Abstract])) OR (ilaprazole[Title/Abstract])	46,659
#3	(((((((((((Peptic Ulcer[MeSH Terms]) OR (Peptic Ulcers[Title/Abstract])) OR (Ulcer, Peptic[Title/Abstract])) OR (Ulcers, Peptic[Title/Abstract])) OR (Ulcers, Peptic[Title/Abstract])) OR (Gastroduodenal Ulcer[Title/Abstract])) OR (Gastroduodenal Ulcers[Title/Abstract])) OR (Ulcer, Gastroduodenal[Title/Abstract])) OR (Ulcers, Gastroduodenal[Title/Abstract])) OR (Marginal Ulcer[Title/Abstract])) OR (Marginal Ulcers[Title/Abstract])) OR (Ulcer, Marginal[Title/Abstract])) OR (Ulcers, Marginal[Title/Abstract])	83,630
#4	(((((((Duodenal Ulcer[MeSH Terms]) OR (Duodenal Ulcers[Title/Abstract])) OR (Ulcer, Duodenal[Title/Abstract])) OR (Ulcers, Duodenal[Title/Abstract])) OR (Curling Ulcer[Title/Abstract])) OR (Ulcer, Curling[Title/Abstract])) OR (Curling's Ulcer[Title/Abstract])) OR (Curlings Ulcer[Title/Abstract])	27,530
#5	(((((((Stomach Ulcer[MeSH Terms]) OR (Stomach Ulcers[Title/Abstract])) OR (Ulcer, Stomach[Title/Abstract])) OR (Ulcers, Stomach[Title/Abstract])) OR (Gastric Ulcer[Title/Abstract])) OR (Gastric Ulcers[Title/Abstract])) OR (Ulcer, Gastric[Title/Abstract])) OR (Ulcers, Gastric[Title/Abstract])	33,640
#6	(((((((((((Randomized Controlled Trial[Publication Type])) OR (Controlled Clinical Trial[Publication Type])) OR (randomized[Title/Abstract])) OR (randomly[Title/Abstract])) OR (random*[Title/Abstract])) OR (double-blind[Title/Abstract])) OR (single-blind[Title/Abstract])) OR (clinical trial*[Title/Abstract])) OR (trial*[Title/Abstract])) OR (RCT[Title/Abstract])) AND (humans[Filter])	1,812,539
#7	#1 OR #2	47,050
#8	#3 OR #4 OR #5	89,257
#9	#6 AND #7 AND #8	1,601

(2) Search Strategy for EMBASE

ID	Search	Results
#1	('potassium competitive acid blocker*' or 'p-cab' or 'vonoprazan' or 'tak?438' or 'tegoprazan' or 'CJ-12420' or 'revaprazan' or 'keverprazan' or 'KFP-H008' or 'soraprazan' or 'linaprazan' or 'AZD-0865').mp. [mp=title, abstract, heading word, drug trade name, original title, device manufacturer, drug manufacturer, device trade name, keyword heading word, floating subheading word, candidate term word]	1840
#2	('proton pump inhibitor' or 'PPI' or 'omeprazole' or 'esomeprazole' or 'lansoprazole' or 'rabeprazole' or 'pantoprazole' or 'dexlansoprazole' or 'ilaprazole').mp. [mp=title, abstract, heading word, drug trade name, original title, device manufacturer, drug manufacturer, device trade name, keyword heading word, floating subheading word, candidate term word]	135623
#3	('Peptic Ulcer' or 'Peptic Ulcers' or 'Ulcer, Peptic' or 'Ulcers, Peptic' or 'Ulcers, Peptic' or 'Gastroduodenal Ulcer' or 'Gastroduodenal Ulcers' or 'Ulcer, Gastroduodenal' or 'Ulcers, Gastroduodenal' or 'Marginal Ulcer' or 'Marginal Ulcers' or 'Ulcer, Marginal' or 'Ulcers, Marginal').mp. [mp=title, abstract, heading word, drug trade name, original title, device manufacturer, drug manufacturer, device trade name, keyword heading word, floating subheading word, candidate term word]	54923
#4	('Duodenal Ulcer' or 'Duodenal Ulcers' or 'Ulcer, Duodenal' or 'Ulcers, Duodenal' or 'Curling Ulcer' or 'Ulcer, Curling' or 'Curling's Ulcer' or 'Curlings Ulcer').mp. [mp=title, abstract, heading word, drug trade name, original title, device manufacturer, drug manufacturer, device trade name, keyword heading word, floating subheading word, candidate term word]	21917
#5	('Stomach Ulcer' or 'Stomach Ulcers' or 'Ulcer, Stomach' or 'Ulcers, Stomach' or 'Gastric Ulcer' or 'Gastric Ulcers' or 'Ulcer, Gastric' or 'Ulcers, Gastric').mp. [mp=title, abstract, heading word, drug trade name, original title, device manufacturer, drug manufacturer, device trade name, keyword heading word, floating subheading word, candidate term word]	45898
#6	('Randomized Controlled Trial' or 'Controlled Clinical Trial' or 'randomized' or 'randomly' or 'random*' or 'double-blind' or 'single-blind' or 'clinical trial*' or 'trial*' or 'RCT').mp. [mp=title, abstract, heading word, drug trade name, original title, device manufacturer, drug manufacturer, device trade name, keyword heading word, floating subheading word, candidate term word]	4070914
#7	#1 OR #2	136175
#8	#3 OR #4 OR #5	101792
#9	#6 AND #7 AND #8	5119

(3) Search Strategy for Web of Science

ID	Search	Results
#1	TS= (vonoprazan OR tak?438 OR tak438 OR tegoprazan OR CJ-12420 OR revaprazan OR keverprazan OR KFP-H008 OR soraprazan OR linaprazan OR AZD-0865 OR potassium competitive acid blocker* OR potassium-competitive acid blockers OR p-cab*) and Preprint Citation Index (Exclude – Database)	2289
#2	TS= (proton pump inhibitor OR PPI OR omeprazole OR esomeprazole OR lansoprazole OR rabeprazole OR pantoprazole OR dexlansoprazole OR ilaprazole) and Preprint Citation Index (Exclude – Database)	108216
#3	TS= (Peptic Ulcer OR Peptic Ulcers OR Ulcer, Peptic OR Ulcers, Peptic OR Ulcers, Peptic OR Gastroduodenal Ulcer OR Gastroduodenal Ulcers OR Ulcer, Gastroduodenal OR Ulcers, Gastroduodenal OR Marginal Ulcer OR Marginal Ulcers OR Ulcer, Marginal OR Ulcers, Marginal) and Preprint Citation Index (Exclude – Database)	80294
#4	TS= (Duodenal Ulcer OR Duodenal Ulcers OR Ulcer, Duodenal OR Ulcers, Duodenal OR Curling Ulcer OR Ulcer, Curling OR Curling's Ulcer OR Curlings Ulcer) and Preprint Citation Index (Exclude – Database)	51714
#5	TS= (Stomach Ulcer OR Stomach Ulcers OR Ulcer, Stomach OR Ulcers, Stomach OR Gastric Ulcer OR Gastric Ulcers OR Ulcer, Gastric OR Ulcers, Gastric) and Preprint Citation Index (Exclude – Database)	104525
#6	TS= (Randomized Controlled Trial OR Controlled Clinical Trial OR randomized OR randomly OR random* OR double-blind OR single-blind OR clinical trial* OR trial* OR RCT) and Preprint Citation Index (Exclude – Database)	595174 4
#7	#1 OR #2	109516
#8	#3 OR #4 OR #5	152496
#9	#6 AND #7 AND #8	5348

(4) Search Strategy for Cochrane Library

ID	Search	Results
#1	(vonoprazan OR tak?438 OR tak438 OR tegoprazan OR CJ-12420 OR revaprazan OR keverprazan OR KFP-H008 OR soraprazan OR linaprazan OR AZD-0865 OR potassium competitive acid blocker* OR potassium-competitive acid blockers OR p-cab*) in Title Abstract Keyword - in Trials (Word variations have been searched)	617
#2	(proton pump inhibitor OR PPI OR omeprazole OR esomeprazole OR lansoprazole OR rabeprazole OR pantoprazole OR dexlansoprazole OR ilaprazole) in Title Abstract Keyword - in Trials (Word variations have been searched)	12121
#3	(Peptic Ulcer OR Peptic Ulcers OR Ulcer, Peptic OR Ulcers, Peptic OR Ulcers, Peptic OR Gastroduodenal Ulcer OR Gastroduodenal Ulcers OR Ulcer, Gastroduodenal OR Ulcers, Gastroduodenal OR Marginal Ulcer OR Marginal Ulcers OR Ulcer, Marginal OR Ulcers, Marginal) in Title Abstract Keyword - in Trials (Word variations have been searched)	5183
#4	(Duodenal Ulcer OR Duodenal Ulcers OR Ulcer, Duodenal OR Ulcers, Duodenal OR Curling Ulcer OR Ulcer, Curling OR Curling's Ulcer OR Curlings Ulcer) in Title Abstract Keyword - in Trials (Word variations have been searched)	4290
#5	(Stomach Ulcer OR Stomach Ulcers OR Ulcer, Stomach OR Ulcers, Stomach OR Gastric Ulcer OR Gastric Ulcers OR Ulcer, Gastric OR Ulcers, Gastric) in Title Abstract Keyword - in Trials (Word variations have been searched)	6117
#6	(Randomized Controlled Trial OR Controlled Clinical Trial OR randomized OR randomly OR random* OR double-blind OR single-blind OR clinical trial* OR trial* OR RCT) in Title Abstract Keyword - in Trials (Word variations have been searched)	1518304
#7	#1 OR #2	12305
#8	#3 OR #4 OR #5	11052
#9	#6 AND #7 AND #8	2752

(5) Search Strategy for CNKI

ID	Search	Results
#1	vonoprazan or tegoprazan or revaprazan or keverprazan	146
#2	proton pump inhibitor or omeprazole or esomeprazole or lansoprazole or rabeprazole or pantoprazole or dexlansoprazole or ilaprazole	35433
#3	peptic ulcer or ulcer or gastric ulcer or duodenal ulcer	59160
#4	#1 or #2	35480
#5	#3 AND #4	1053

(6) Search Strategy for Wanfang database

ID	Search	Results
#1	vonoprazan or tegoprazan or revaprazan or keverprazan	156
#2	proton pump inhibitor or omeprazole or esomeprazole or lansoprazole or rabeprazole or pantoprazole or dexlansoprazole or ilaprazole	53581
#3	peptic ulcer or ulcer or gastric ulcer or duodenal ulcer	81948
#4	#1 or #2	53692
#5	#3 AND #4	3296

Table S3. The basic characteristics of included studies on duodenal ulcer (N=28).

Study ID	Study number	Study design	Country/ Region	Masking	Study period	Participant	Disease	Treatment measure	Age (years, mean±SD)	Sex (male/female)	Treatment duration	Outcome Measures
Hou 2022[1]	Study 2	multicenter	Asia (China and Korea)	Quadruple	2017.04-2019.03	533	DU	VPZ<br>LPZ	42.0±12.2<br>41.4±12.9	166/99<br>176/92	6 weeks	abde
Miwa 2017a[2]	Study 3	multicenter	Japan	Quadruple	2011.10-2013.02	372	DU	VPZ<br>LPZ	49.9±14.6<br>50.2±14.8	125/59<br>120/68	6 weeks	acdef
Tan 2022[3]	Study 6	multicenter	China	double	2019.09-2020.03	105	DU	KPZ<br>LPZ	44.4±12.4<br>43.5±12.3	41/20<br>23/21	6 weeks	ade
Tan 2023[4]	Study 7	multicenter	China	double	2020.09-2021.03	358	DU	KPZ<br>LPZ	44.2±12.4<br>44.4±11.8	111/69<br>108/70	6 weeks	acdef
Avner 1995[5]	Study 8	multicenter	USA	double	NA	150	DU	LPZ<br>PLA	41.3±12.8<br>44.7±13.0	44/31<br>55/20	4 weeks	a
Lanza 1994[6]	Study 9	multicenter	USA	double	NA	200	DU	LPZ<br>PLA	NA	NA	4 weeks	a
Graham 1990[7]	Study 10	multicenter	USA	double	NA	147	DU	OPZ<br>PLA	47.5<br>51.0	75/24<br>36/12	4 weeks	ae
Colin 2002[8]	Study 11	multicenter	France	double	NA	150	DU	OPZ<br>PLA	64.0<br>67.0	NA	3 weeks	abc
Catalano 2000[9]	Study 13	single center	Italy	double	NA	172	DU	PPZ<br>OPZ	43.2±10.1<br>44.7±12.1	40/44<br>46/42	1 week	abc
Chang 1995[10]	Study 14	single center	Taiwan	double	NA	111	DU	LPZ<br>OPZ	56.4±13.9<br>59.3±12.8	53/4<br>50/4	4 weeks	ab
Li 2019[11]	Study 15	multicenter	China	double	2008.12-2010.01	259	DU	RPZ<br>IPZ	39.3±11.4<br>37.6±11.1	95/35<br>91/38	4 weeks	acde
Wang 2012a[12]	Study 20	multicenter	China	double	2005.10-2006.01	494	DU	IPZ<br>OPZ	41.2±12.1<br>40.9±11.9	232/98<br>129/35	4 weeks	acdef
Wang 2011[13]	Study 21	multicenter	China	double	2004.11-2005.01	118	DU	IPZ<br>OPZ	39.0±10.8<br>39.2±11.6	38/20<br>45/14	4 weeks	acde
Rehner 1995[14]	Study 22	multicenter	Germany	NA	NA	286	DU	PPZ<br>OPZ	46.4±12.5<br>47.7±12.7	127/66<br>33/60	4 weeks	ade

Ekström 1995[15]	Study 23	multicenter	Sweden	double	1990.02-1991.08	279	DU	LPZ<br>OPZ	54.4<br>55.3	86/57<br>85/51	4 weeks	ae
Dekkers 1999[16]	Study 24	multicenter	Europe	double	NA	205	DU	RPZ<br>OPZ	47.3±13.5<br>47.8±13.2	65/37<br>69/34	4 weeks	ade
Dobrilla 1999[17]	Study 25	multicenter	Europe	double	1993.03-1994.02	251	DU	LPZ<br>OPZ	43.5±12.8<br>44.9±11.5	108/59<br>57/27	4 weeks	ae
Ho 2009b[18]	Study 27	multicenter	Asia	double	2002.09-2004.02	202	DU	IPZ<br>OPZ	47.9±13.8<br>51.6±14.4	70/28<br>78/26	4 weeks	acde
Zhou 2009a[19]	Study 31	multicenter	China	double	2005.10-2006.02	480	DU	IPZ<br>OPZ	NA	NA	4 weeks	adef
Zheng 2002[20]	Study 32	multicenter	China	double	2002.01-2002.07	109	DU	RPZ<br>OPZ	39.9±12.1<br>42.4±13.0	36/17<br>40/16	2 weeks	abce
Zhou 2009b[21]	Study 33	multicenter	China	double	2004.11-2005.02	226	DU	IPZ<br>OPZ	NA	NA	4 weeks	ade
Liu 2017[22]	Study 34	multicenter	China	double	2014.09-2016.09	82	DU	RPZ<br>OPZ	42.6±3.8<br>41.9±4.1	26/15<br>29/12	2 weeks	abc
Shi 2019[23]	Study 39	single center	China	NA	2015.01-2018.08	206	DU	IPZ<br>OPZ	40.2±3.6<br>39.6±3.9	64/39<br>59/44	4 weeks	abce
Deng 2009[24]	Study 42	single center	China	NA	2007.08-2008.07	124	DU	EPZ<br>OPZ	NA	NA	1 week	abcde
Zou 2014[25]	Study 44	single center	China	NA	2011.08-2012.08	134	DU	EPZ<br>OPZ	35.7±6.7<br>35.4±5.5	42/25<br>38/29	10 d	abc
Fang 2016[26]	Study 46	single center	China	NA	2013.09-2015.02	70	DU	EPZ<br>OPZ	45.5±12.8<br>47.0±12.5	24/11<br>23/12	4 weeks	ade
Xia 2014[27]	Study 55	single center	China	NA	2010.02-2013.10	67	DU	EPZ<br>OPZ	43.7±8.2<br>42.3±7.8	22/13<br>20/12	4 weeks	abc
Zhang 2011[28]	Study 56	single center	China	double	2008.09-2010.08	90	DU	EPZ<br>OPZ	NA	NA	4 weeks	abce

DU: duodenal ulcer; NA: not applicable; a: ulcer healing rate; b: *Helicobacter pylori* (*H.pylori*) eradication rate; c: ulcer healing rate in *H.pylori* positive or negative patients; d: remission rate of ulcer-related symptoms; e: adverse events; f: ulcer healing rate of different genotypes; VPZ: vonoprazan; TPZ: tegoprazan; KPZ: keverprazan; OPZ: omeprazole; PPZ: pantoprazole; LPZ: lansoprazole; RPZ: rabeprazole; EPZ: esomeprazole; IPZ: ilaprazole; PLA: placebo.

Table S4. The basic characteristics of included studies on gastric ulcer (N=10).

Study ID	Study number	Study design	Country/ Region	Masking	Study period	Participant	Disease	Treatment measure	Age (years, mean±SD)	Sex (male/female)	Treatment duration	Outcome Measures
NCT03050307[29]	Study 1	multicenter	Asia (China and Korea)	Quadruple	2017.04-2020.02	234	GU	VPZ<br>LPZ	54.0±13.7<br>53.5±13.4	86/29<br>92/27	8 weeks	abde
Miwa 2017b[2]	Study 4	multicenter	Japan	Quadruple	2011.11-2012.12	482	GU	VPZ<br>LPZ	58.2±13.2<br>58.6±13.5	163/81<br>170/68	8 weeks	acdef
Cho 2020[30]	Study 5	multicenter	Korea	Quadruple	2016.05-2018.07	173	GU	TPZ<br>LPZ	53.4<br>54.2	58/30<br>47/38	8 weeks	ace
Ando 2005[31]	Study 12	multicenter	NA	double	NA	80	GU	RPZ<br>OPZ	51.6±10.4<br>50.6±8.0	31/8<br>33/8	8 weeks	af
Ho 2009a[18]	Study 26	multicenter	Asia	double	2002.09-2004.02	142	GU	IPZ<br>OPZ	54.3±13.1<br>52.9±15.4	44/28<br>46/24	4 weeks	acde
Chen 2021[32]	Study 35	single center	China	NA	2019.01-2020.06	100	GU	VPZ<br>LPZ	42.9±8.1<br>44.1±8.7	31/22<br>28/19	2 weeks	abce
Xiao 2014[33]	Study 45	single center	China	NA	2012.03-2013.03	100	GU	PPZ<br>RPZ	46.9±8.3<br>46.5±8.2	28/22<br>25/25	4 weeks	abc
Chen 2018[34]	Study 47	single center	China	NA	2016.09-2018.03	150	GU	EPZ<br>RPZ<br>OPZ	41.5±10.9<br>41.7±8.9<br>42.1±10.0	26/24<br>25/25<br>27/23	4 weeks	abce
Kong 2019[35]	Study 49	single center	China	NA	2017.01-2018.06	90	GU	PPZ<br>OPZ	40.3±5.2<br>39.8±5.2	30/15<br>31/14	4 weeks	ae
Liao 2007[36]	Study 52	single center	China	NA	NA	265	GU	OPZ<br>LPZ<br>EPZ<br>PPZ<br>RPZ	42<br>39<br>41<br>40<br>38.5	38/22<br>30/19<br>30/24<br>36/19<br>26/21	3 weeks	abce

GU: gastric ulcer; NA: not applicable; a: ulcer healing rate; b: *Helicobacter pylori* (*H. pylori*) eradication rate; c: ulcer healing rate in *H. pylori* positive or negative patients; d: remission rate of ulcer-related symptoms; e: adverse events; f: ulcer healing rate of different genotypes; VPZ: vonoprazan; TPZ: tegoprazan; KPZ: keverprazan; OPZ: omeprazole; PPZ: pantoprazole; LPZ: lansoprazole; RPZ: rabeprazole; EPZ: esomeprazole; IPZ: ilaprazole; PLA: placebo.

Table S5. The basic characteristics of included studies on duodenal ulcer and gastric ulcer (N=6).

Study ID	Study number	Study design	Country/ Region	Masking	Study period	Participant	Disease	Treatment measure	Age (years, mean±SD)	Sex (male/female)	Treatment duration	Outcome Measures
Hawkey 2003[37]	Study 17	multicenter	Europe	double	NA	173	DU+GU	RPZ<br>OPZ	49.4±14.3<br>53.7±15.1	57/30<br>50/36	1 week	ace
Ji 2006[38]	Study 18	multicenter	Korea	NA	2002.06-2003.10	112	DU+GU	RPZ<br>OPZ	49.4±14.3<br>51.9±13.0	42/14<br>38/18	6 weeks	adf
Spinzi 1998[39]	Study 19	multicenter	Italy	single	1996.06-1997.02	356	DU+GU	OPZ<br>LPZ	49.3±15.3<br>50.1±15.5	109/61<br>120/66	1 week	abce
Zeng 2001[40]	Study 30	multicenter	China	open	1999.06-1999.09	137	DU+GU	RPZ<br>OPZ	NA	NA	4 weeks/<br>6 weeks	ae
Lin 2001[41]	Study 41	multicenter	China	NA	1999.07-1999.11	118	DU+GU	PPZ<br>OPZ	45.4±11.7<br>47.1±13.8	47/13<br>39/19	4 weeks/<br>6 weeks	ae
Liu 2005[42]	Study 53	single center	China	NA	2001.01-2003.06	96	DU+GU	PPZ<br>OPZ	41.2±11.0<br>43.1±11.8	29/21<br>31/15	4weeks/<br>6weeks	ae

DU: duodenal ulcer; GU: gastric ulcer; NA: not applicable; a: ulcer healing rate; b: *Helicobacter pylori* (*H. pylori*) eradication rate; c: ulcer healing rate in *H. pylori* positive or negative patients; d: remission rate of ulcer-related symptoms; e: adverse events; f: ulcer healing rate of different genotypes; VPZ: vonoprazan; TPZ: tegoprazan; KPZ: keverprazan; OPZ: omeprazole; PPZ: pantoprazole; LPZ: lansoprazole; RPZ: rabeprazole; EPZ: esomeprazole; IPZ: ilaprazole; PLA: placebo.

Table S6. The basic characteristics of included studies on peptic ulcer with unspecified location (N=12).

Study ID	Study number	Study design	Country/ Region	Masking	Study period	Participant	Disease	Treatment measure	Age (years, mean±SD)	Sex (male/female)	Treatment duration	Outcome Measures
Feng 2005[43]	Study 16	single center	Japan	double	1997.05-2003.05	40	PU	LPZ<br>PLA	41.6±1.2<br>38.8±1.8	21/4<br>13/2	10d	ac
Wang 2022[44]	Study 28	single center	China	double	2021.10-2022.02	84	PU	VPZ<br>LPZ	42.6±5.9<br>39.7±7.7	20/22<br>21/21	4 weeks	abce
Huang 2023[45]	Study 29	single center	China	double	2020.01-2021.07	220	PU	VPZ<br>LPZ	44.3±2.6<br>45.2±2.6	54/56<br>56/54	4-8weeks	ace
Qin 2023[46]	Study 36	single center	China	NA	2020.01-2022.06	72	PU	VPZ<br>RPZ	40.7±7.8<br>42.6±5.2	17/19<br>20/16	2 weeks	abce
Yu 2023[47]	Study 37	single center	China	NA	2020.01-2022.12	82	PU	VPZ<br>OPZ	36.9±8.2<br>37.7±8.1	21/20<br>19/22	4 weeks	ace
Chen 2020[48]	Study 38	single center	China	NA	2018.10-2019.06	70	PU	PPZ<br>OPZ	52.5±7.3<br>52.5±7.8	24/11<br>23/12	6 weeks	abc
Zhang 2007[49]	Study 40	single center	China	NA	2004.12-2007.01	60	PU	EPZ<br>OPZ	NA	NA	1 week	abc
Sun 2011[50]	Study 43	single center	China	NA	2008.09-2010.09	92	PU	RPZ<br>OPZ	37.8±14.5<br>37.9±12.8	26/20<br>25/21	2 weeks	abce
Li 2018[51]	Study 48	single center	China	NA	2016.01-2016.06	52	PU	RPZ<br>OPZ	43.5±1.3<br>43.7±1.5	16/10<br>15/11	4 weeks	abc
Wang 2012b[52]	Study 50	single center	China	NA	2011.01-2011.10	66	PU	EPZ<br>OPZ	NA	NA	4 weeks	ade
Li 2009[53]	Study 51	single center	China	NA	2008.04-2009.02	72	PU	RPZ<br>OPZ	36.5<br>35.5	22/14<br>26/10	4 weeks	abcde
Yang 2000[54]	Study 54	single center	China	NA	1998.02-1999.01	94	PU	LPZ<br>OPZ	45.0±15.6<br>44.5±16.1	40/14<br>33/7	4 weeks	ade

PU: peptic ulcer; NA: not applicable; a: ulcer healing rate; b: *Helicobacter pylori* (*H. pylori*) eradication rate; c: ulcer healing rate in *H. pylori* positive or negative patients; d: remission rate of ulcer-related symptoms; e: adverse events; f: ulcer healing rate of different genotypes; VPZ: vonoprazan; TPZ: tegoprazan; KPZ: keverprazan; OPZ: omeprazole; PPZ: pantoprazole; LPZ: lansoprazole; RPZ: rabeprazole; EPZ: esomeprazole; IPZ: ilaprazole; PLA: placebo.

Table S7. The overall ulcer healing rates of P-CABs and PPIs in the meta-analysis.

Regimen	Number of studies	n/N	Ulcer healing rates, % (95% CI)	Het. I^2 (%)
vonoprazan	9	996/1057	95.5 (94.2-96.7)	46.9
keverprazan	2	223/235	95.0 (92.3-97.8)	0
tegoprazan	1	91/96	94.8 (90.3-99.2)	0
omeprazole	40	2259/2675	84.8 (82.3-87.3)	74.3
lansoprazole	19	1959/2137	91.7 (89.5-93.9)	75.5
pantoprazole	8	514/572	90.9 (88.5-93.2)	8.3
rabeprazole	15	708/809	89.3 (85.2-93.3)	81.7
esomeprazole	9	377/411	92.1 (89.5-94.7)	0
ilaprazole	8	1054/1178	88.7 (84.0-93.4)	88.4
placebo	5	124/245	45.0 (18.1-72.0)	95.7

n: number of patients successfully treated; N: number of included patients; *H. pylori*: *Helicobacter pylori*; Het. I2: Heterogeneity.

Table S8. The overall *H. pylori* eradication rates of P-CABs and PPIs in the meta-analysis.

Regimen	Number of studies	n/N	*H. pylori* eradication rates, % (95% CI)	Het. I^2 (%)
vonoprazan	5	383/417	92.9 (90.4-95.3)	19.3
keverprazan	—	—	—	—
tegoprazan	—	—	—	—
omeprazole	18	827/1061	79.9 (75.4-84.4)	72
lansoprazole	7	523/650	81.4 (75.7-87.0)	68.6
pantoprazole	4	196/224	89.3 (82.2-96.4)	70.1
rabeprazole	9	340/385	89.4 (85.0-93.8)	55.7
esomeprazole	7	310/343	91.2 (88.2-94.2)	0
ilaprazole	1	95/103	92.2 (87.1-97.4)	0
placebo	1	51/67	76.1 (65.9-86.3)	0

n: number of patients successfully treated; N: number of included patients; *H. pylori*: *Helicobacter pylori*; Het. I^2: Heterogeneity.

2 Supplementary Figures

Figure S1. Risk of bias for included studies assessed by Cochrane risk of bias tool (ROB 2.0).

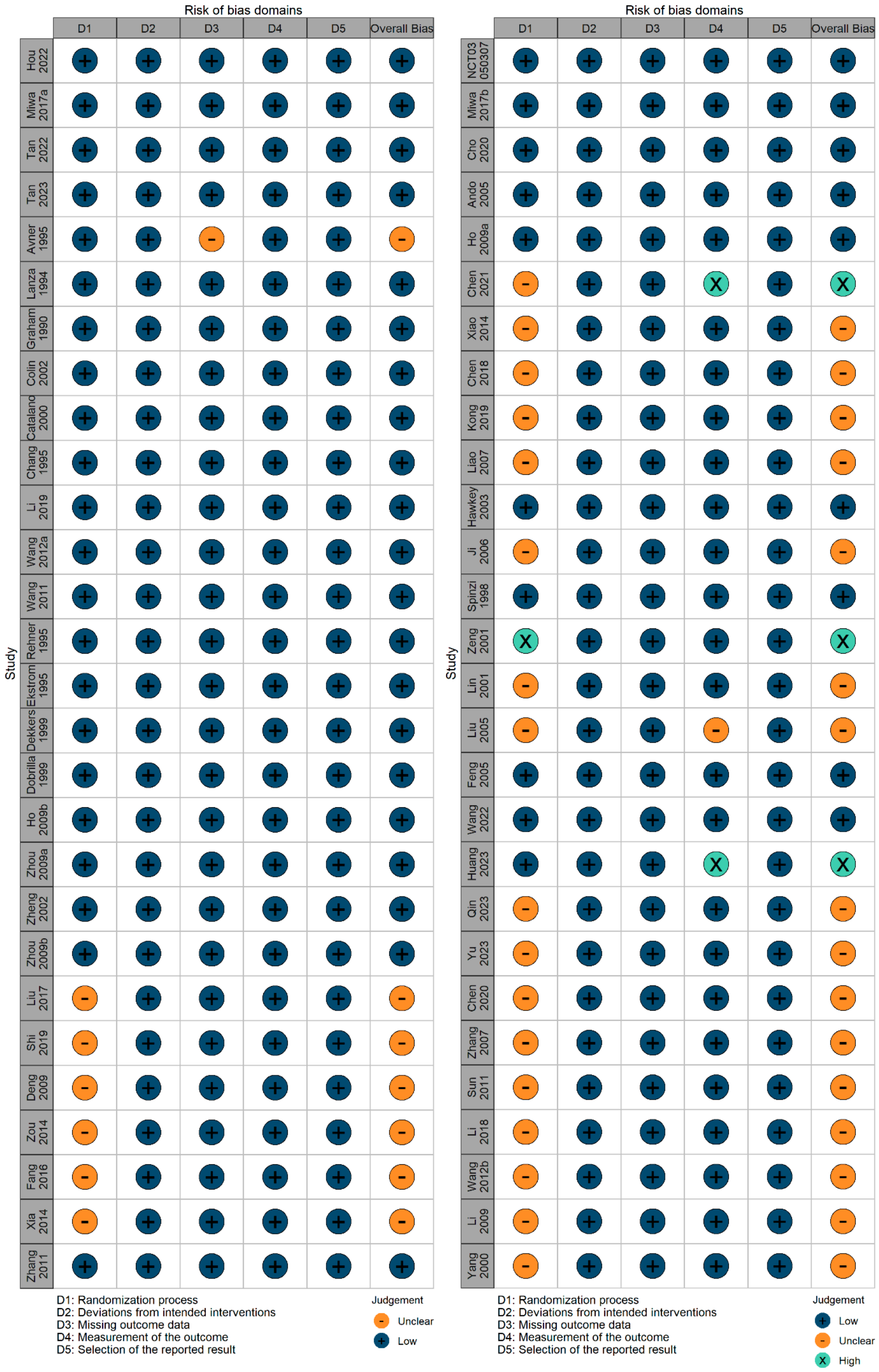

Figure S2. The risk of bias map depicting the proportion of each bias risk item in all studies.

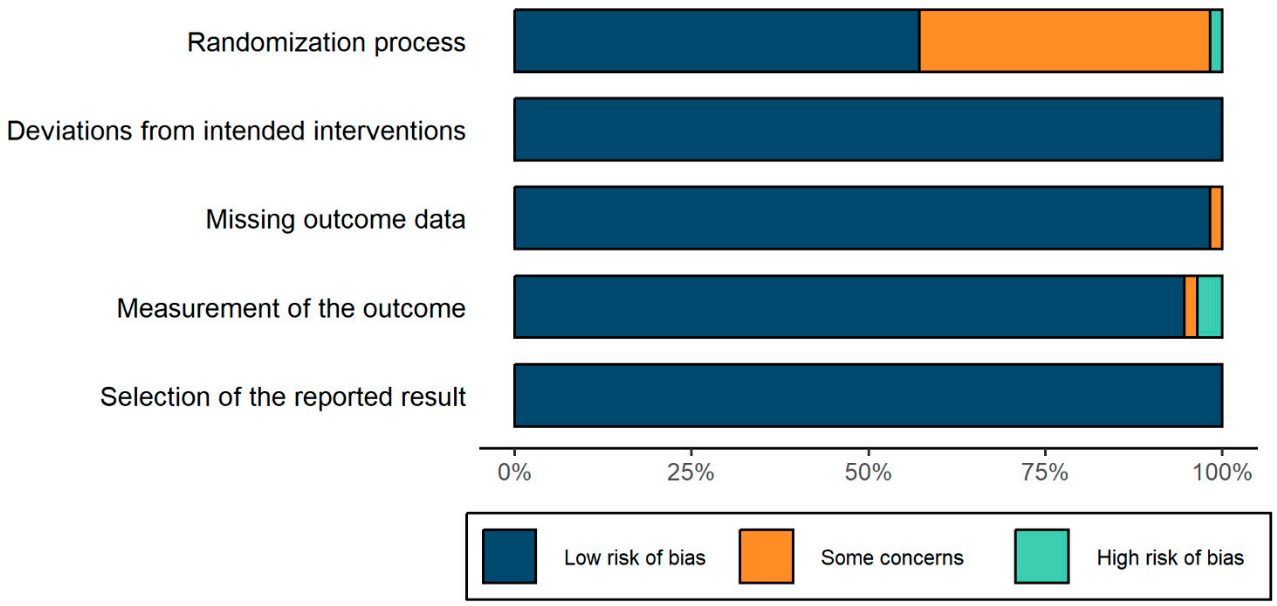

Figure S3. Detection of local inconsistencies in ulcer healing rates using node-splitting method.

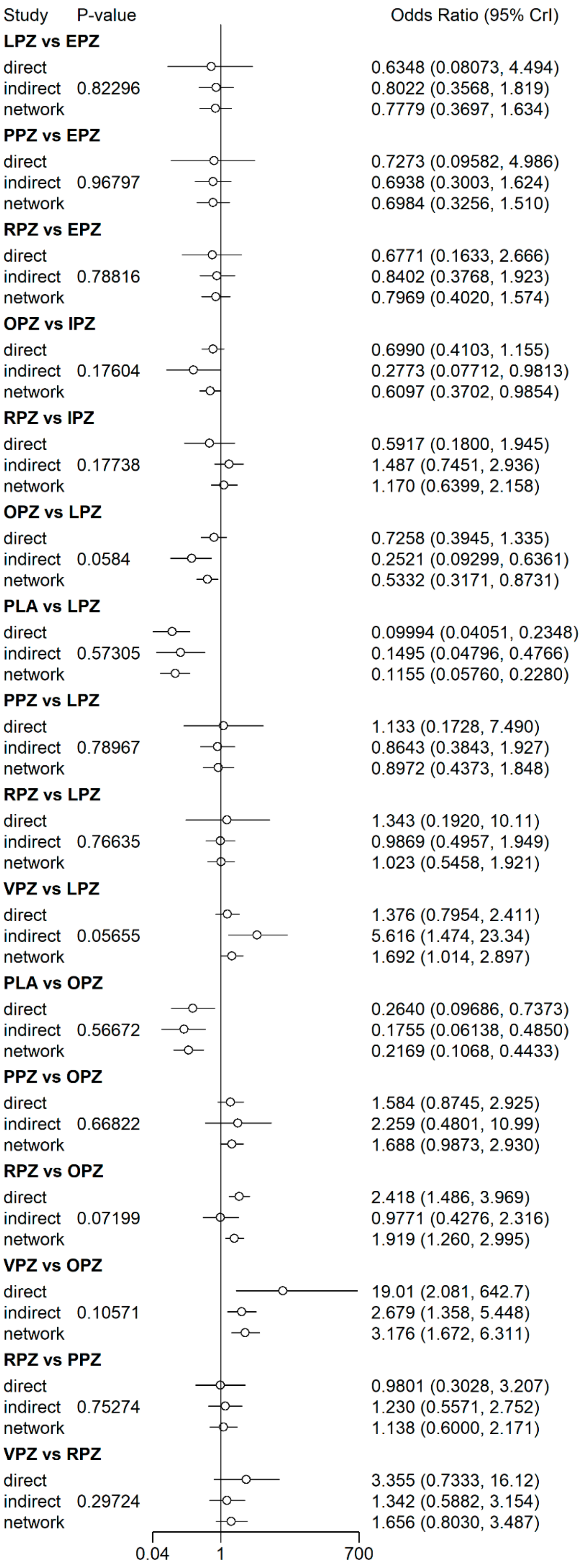

Figure S4. Network forest plot for all direct and mixed comparisons of *H. pylori* eradication rates.

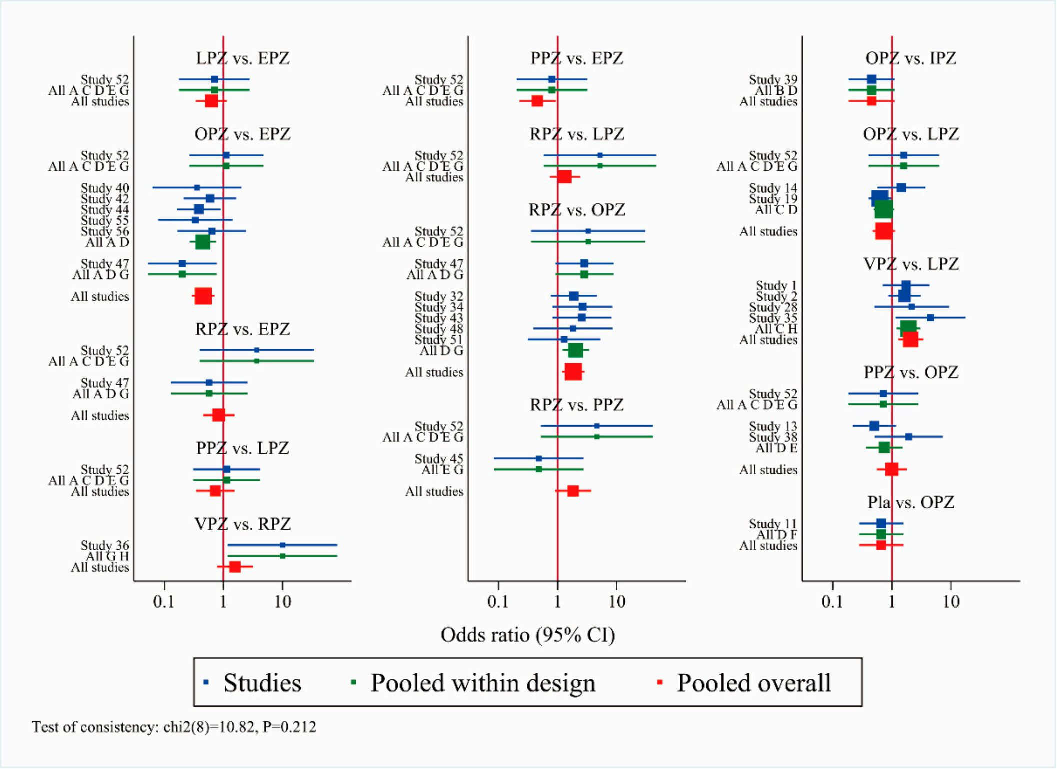

Figure S5. Detection of local inconsistencies in *H. pylori* eradication rates using node-splitting method.

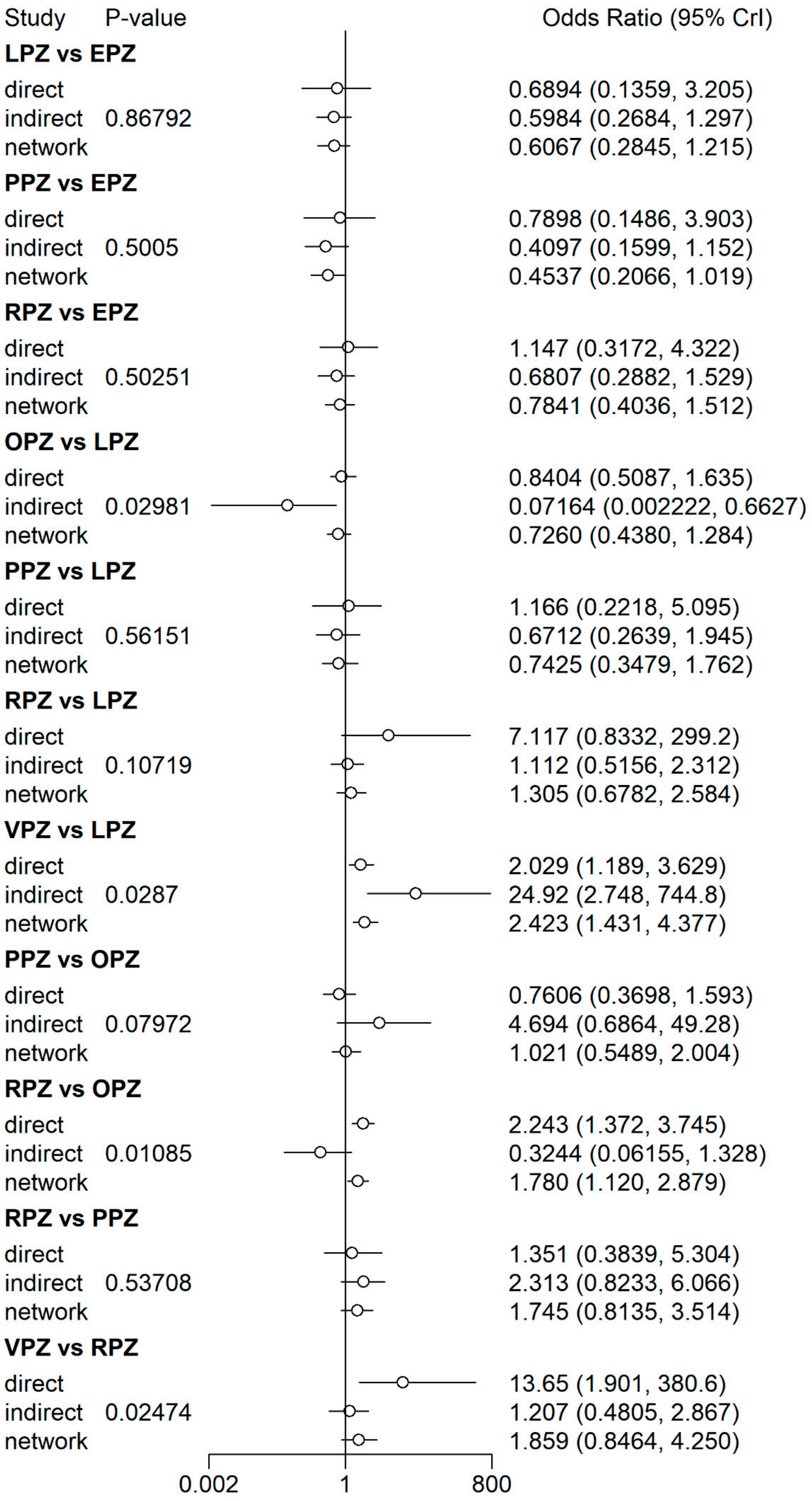

Figure S6. Forest plot of meta-analysis results for abdominal distension remission rates.

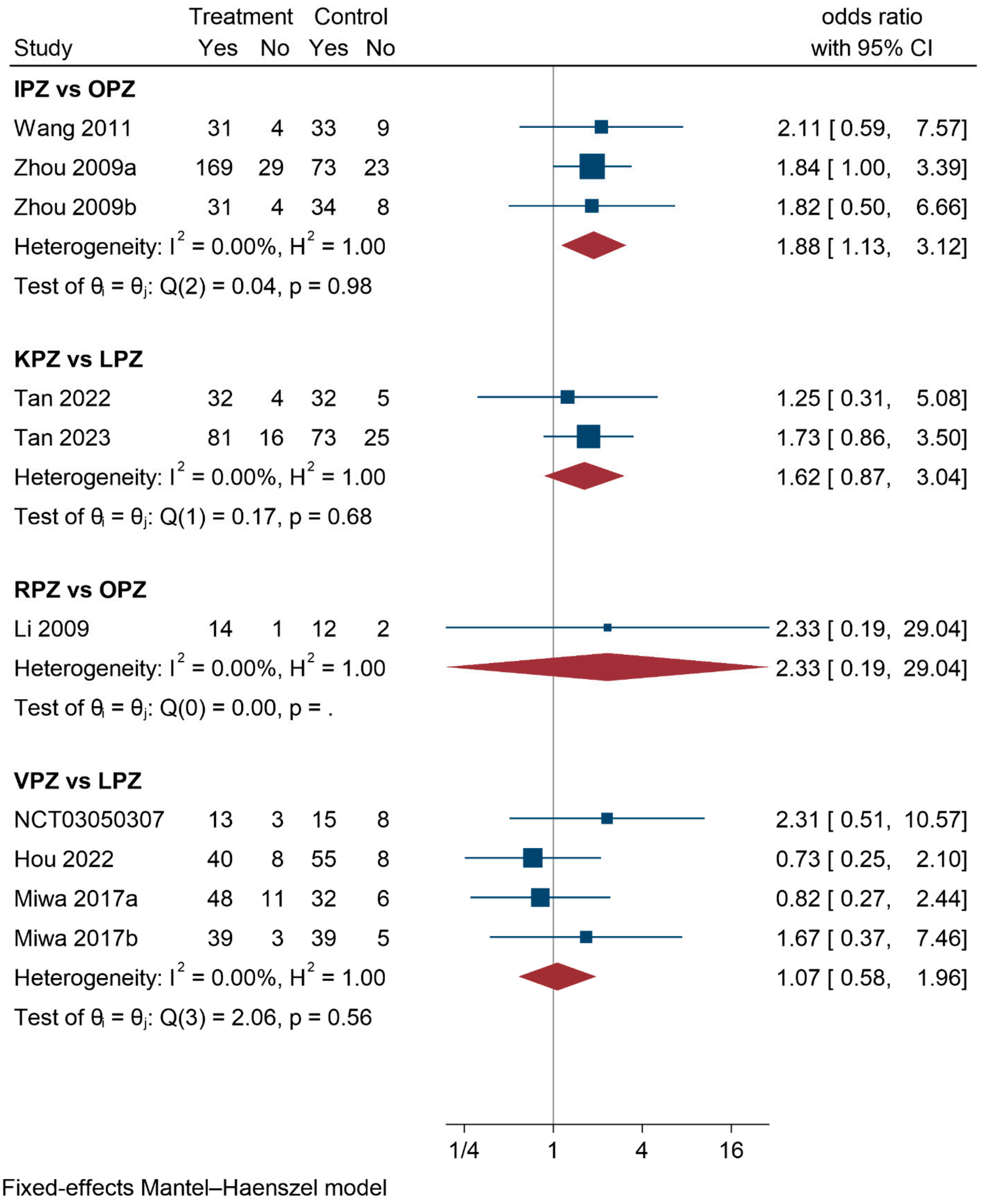

Figure S7. Forest plot of meta-analysis results for nausea and vomiting remission rates.

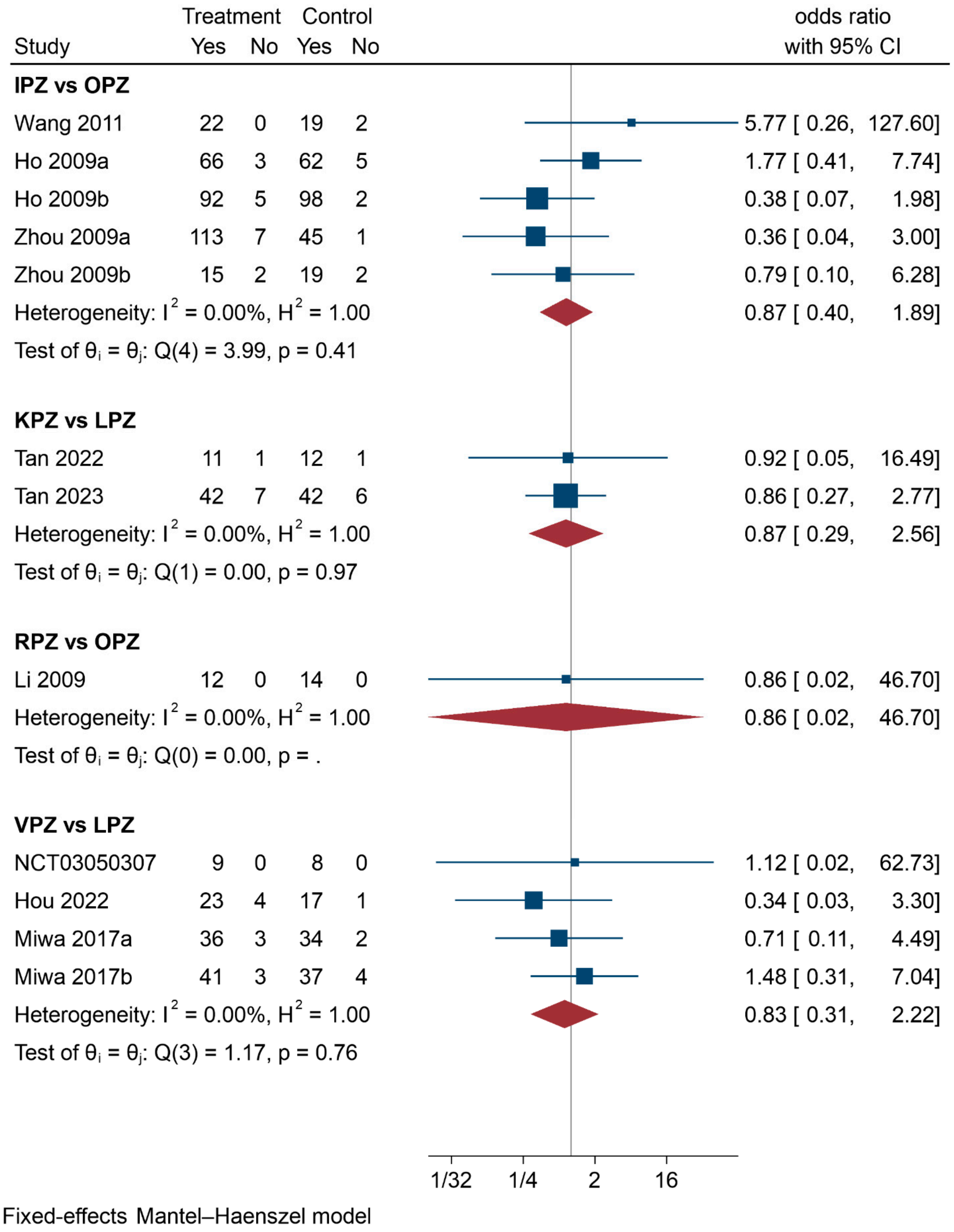

Figure S8. Forest plot of meta-analysis results for heartburn remission rates.

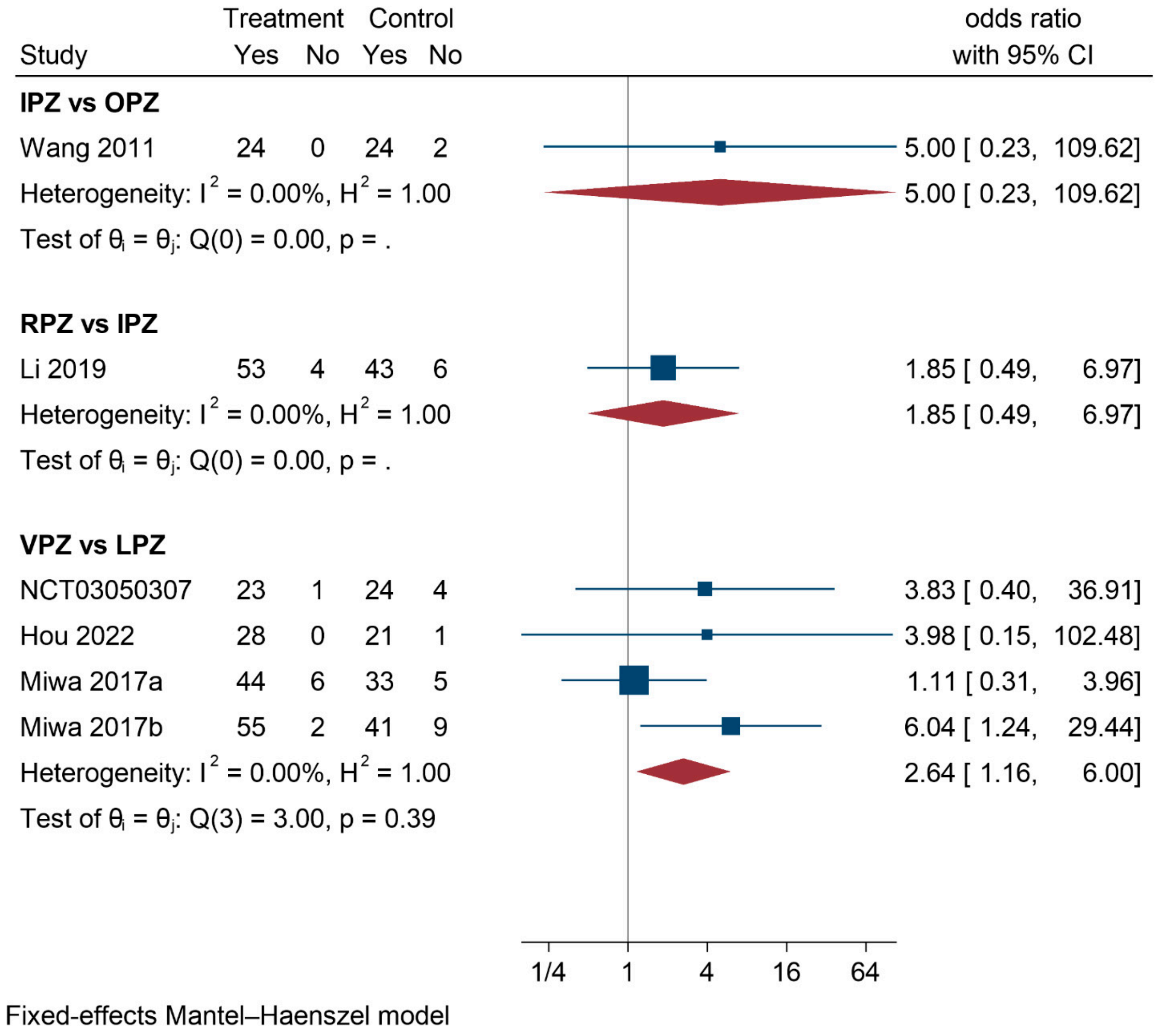

Figure S9. Forest plot of meta-analysis results for regurgitation remission rates.

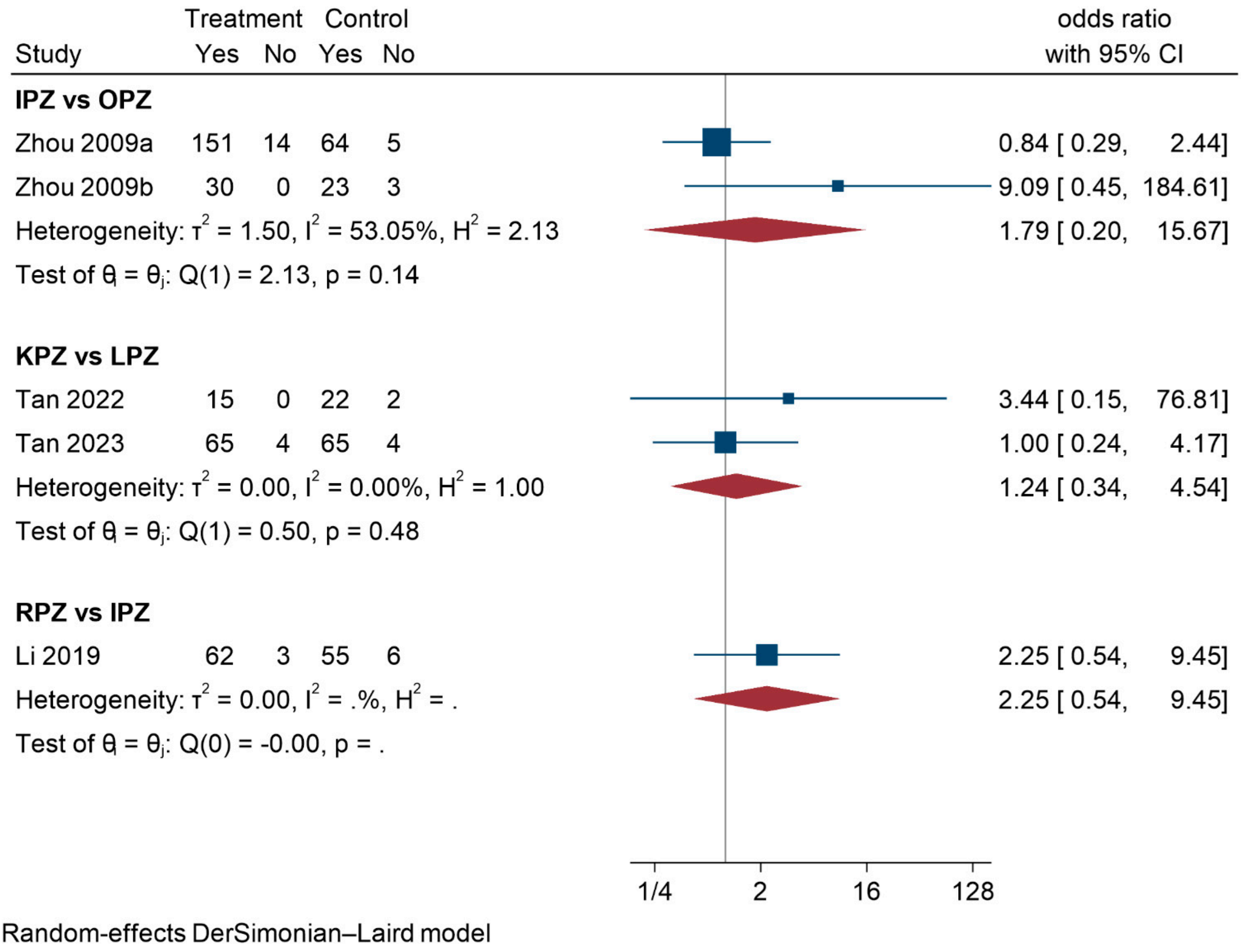

Figure S10. Forest plot of meta-analysis results for epigastric burning sensation remission rates.

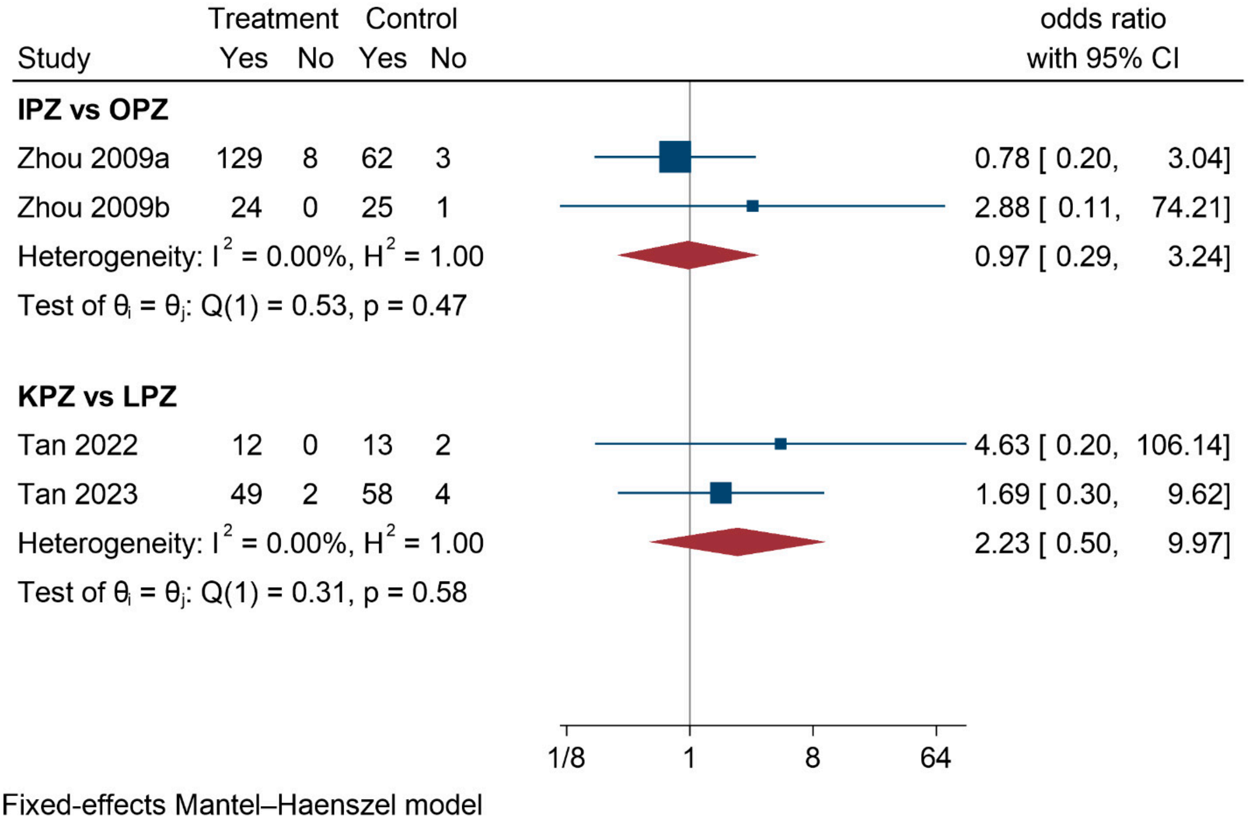

Figure S11. Forest plot of meta-analysis results for lack of appetite remission rates.

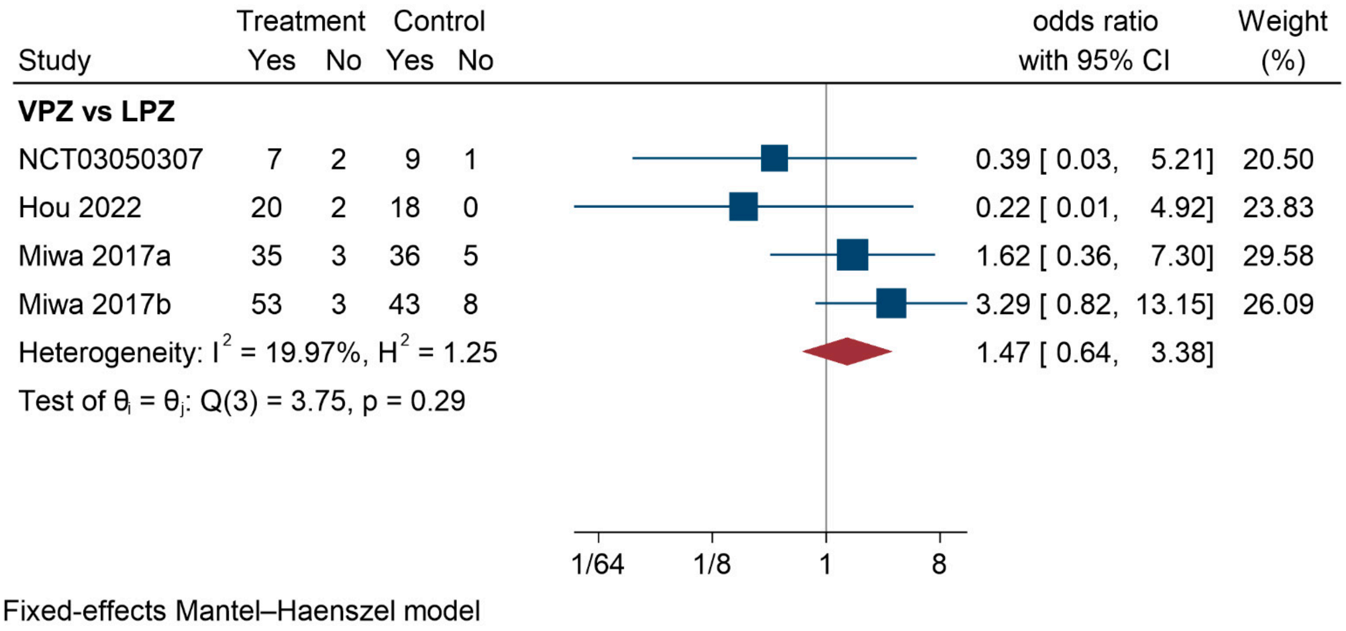

Figure S12. Forest plot of meta-analysis results for belching remission rates.

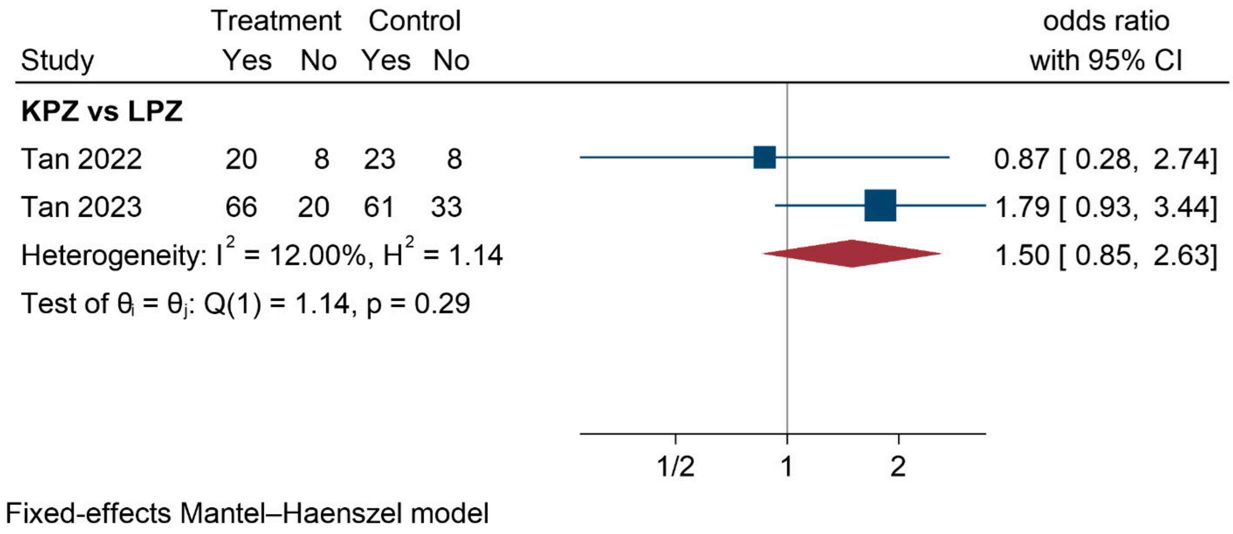

Figure S13. Network forest plot for all direct and mixed comparisons of pain symptom remission rates.

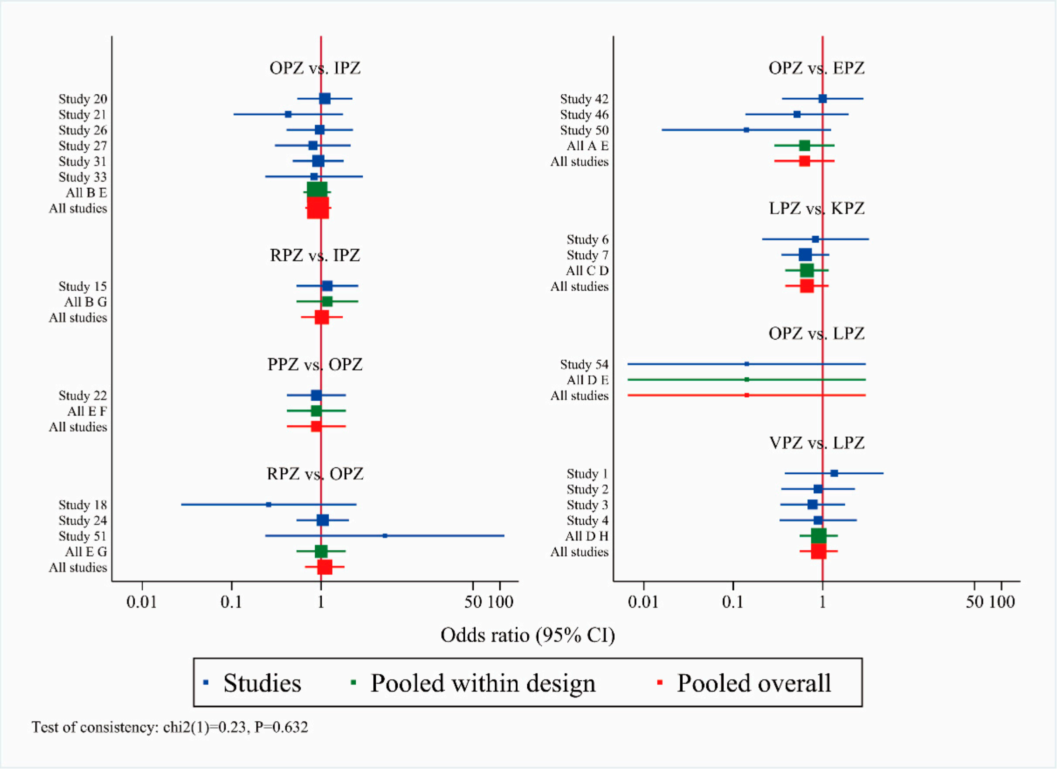

Figure S14. Detection of local inconsistencies in pain symptom remission rates using node-splitting method.

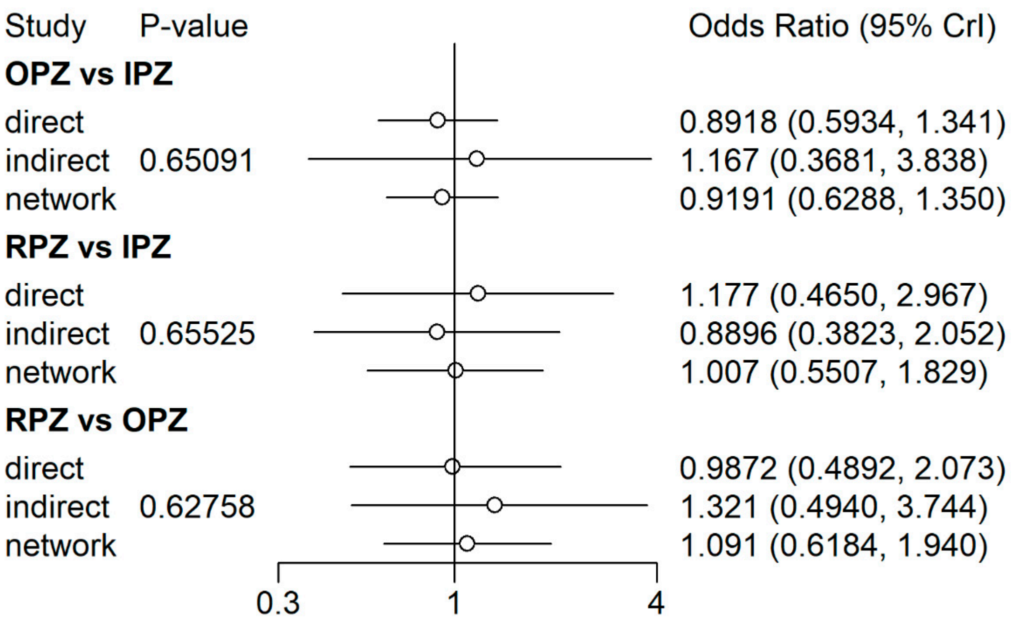

Figure S15. Network forest plot for all direct and mixed comparisons of adverse events.

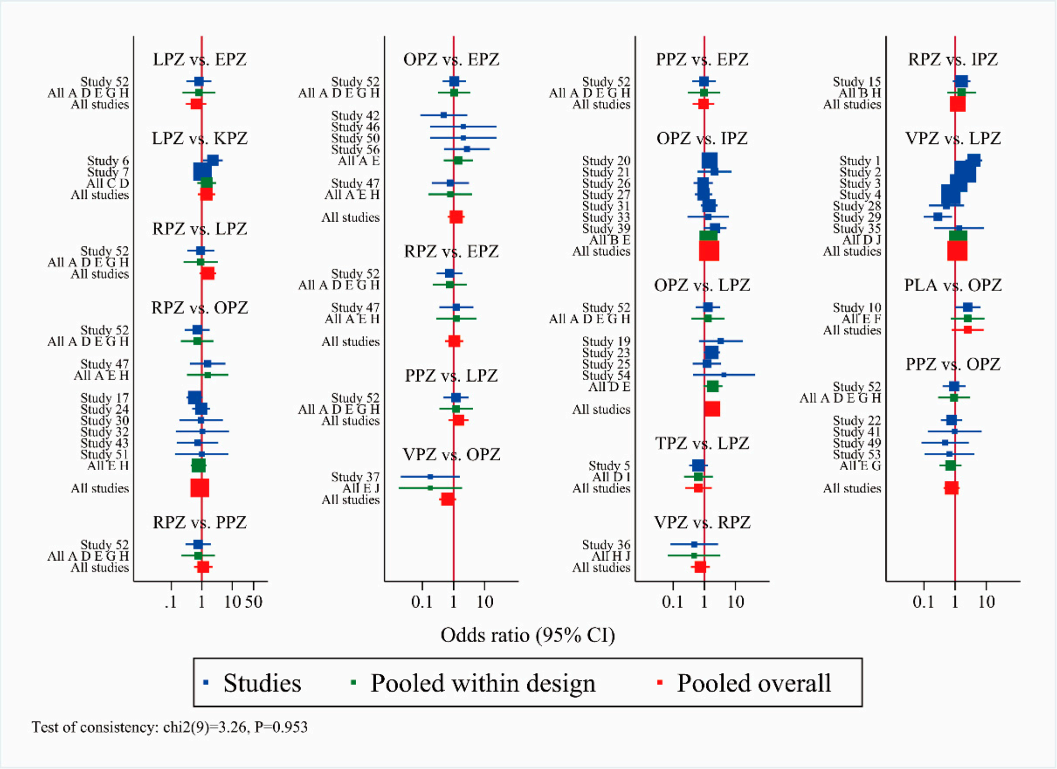

Figure S16. Detection of local inconsistencies in adverse events using node-splitting method.

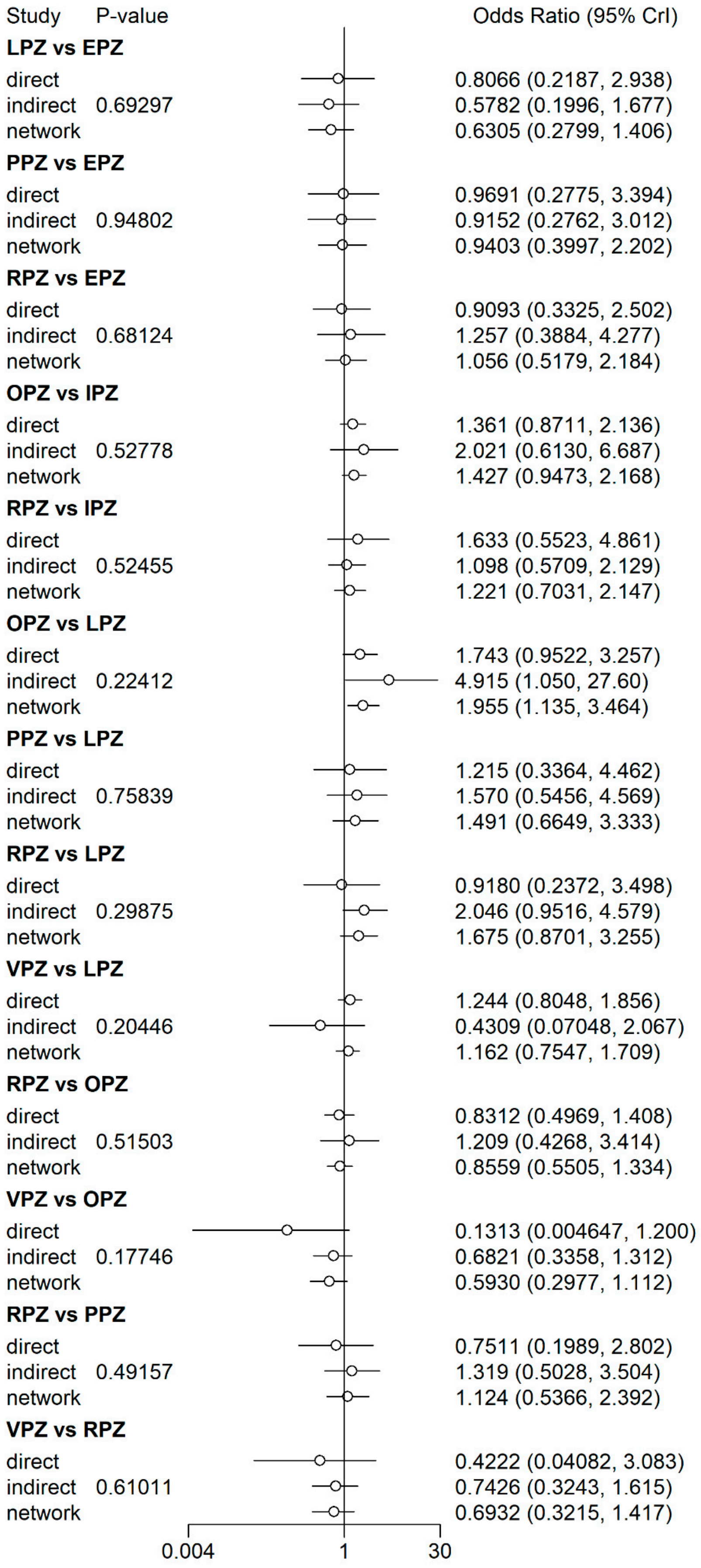

Figure S17. Forest plot of network meta-analysis results of adverse events for P-CABs and PPIs monotherapy or combination antibiotic therapy. (A) P-CABs and PPIs monotherapy; (B) P-CABs and PPIs combination antibiotic therapy.

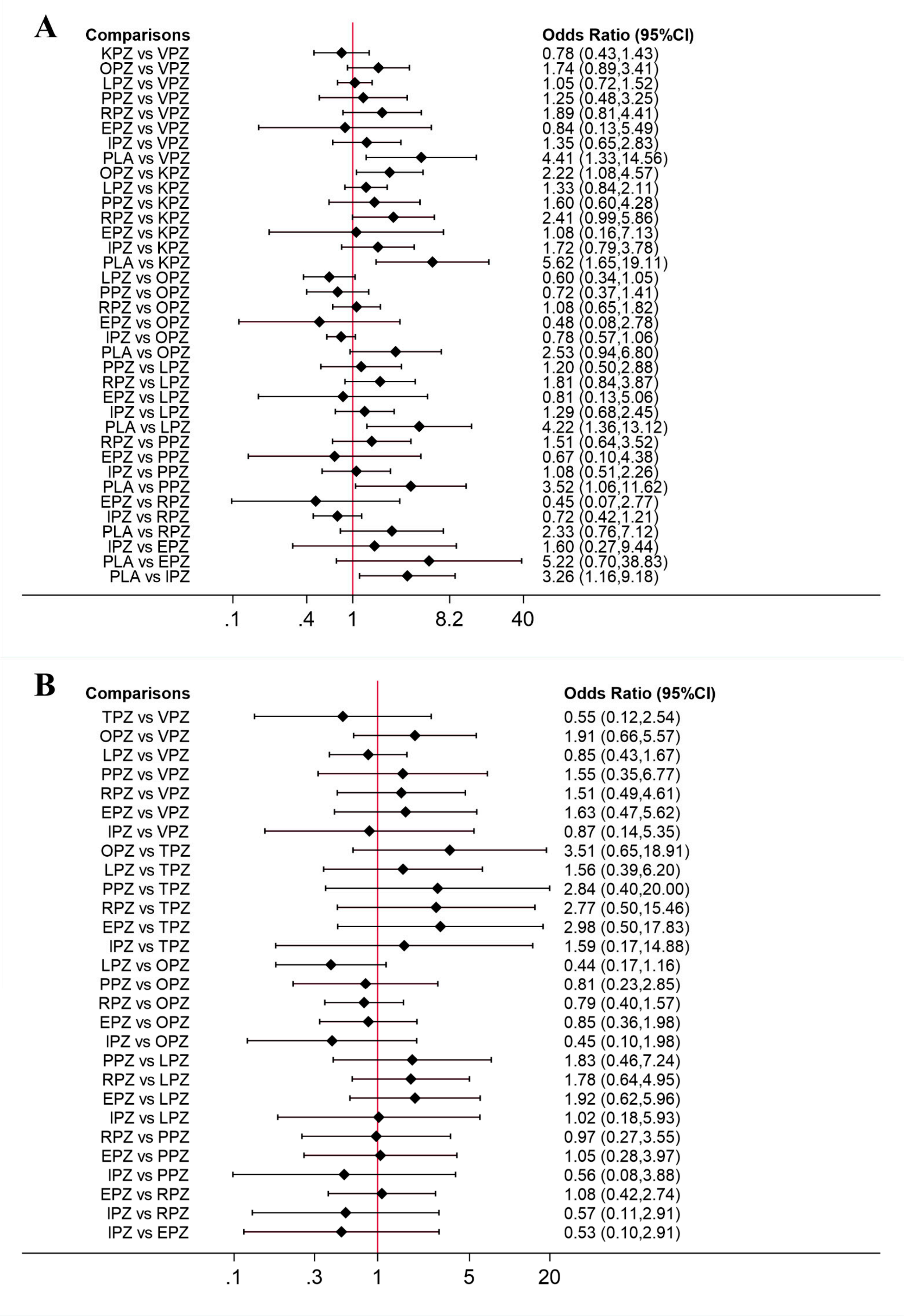

Figure S18. The cumulative rank probability plot of adverse events for P-CABs and PPIs monotherapy or combination antibiotic therapy. (A) P-CAB and PPIs monotherapy; (B) P-CABs and PPIs combination antibiotic therapy.

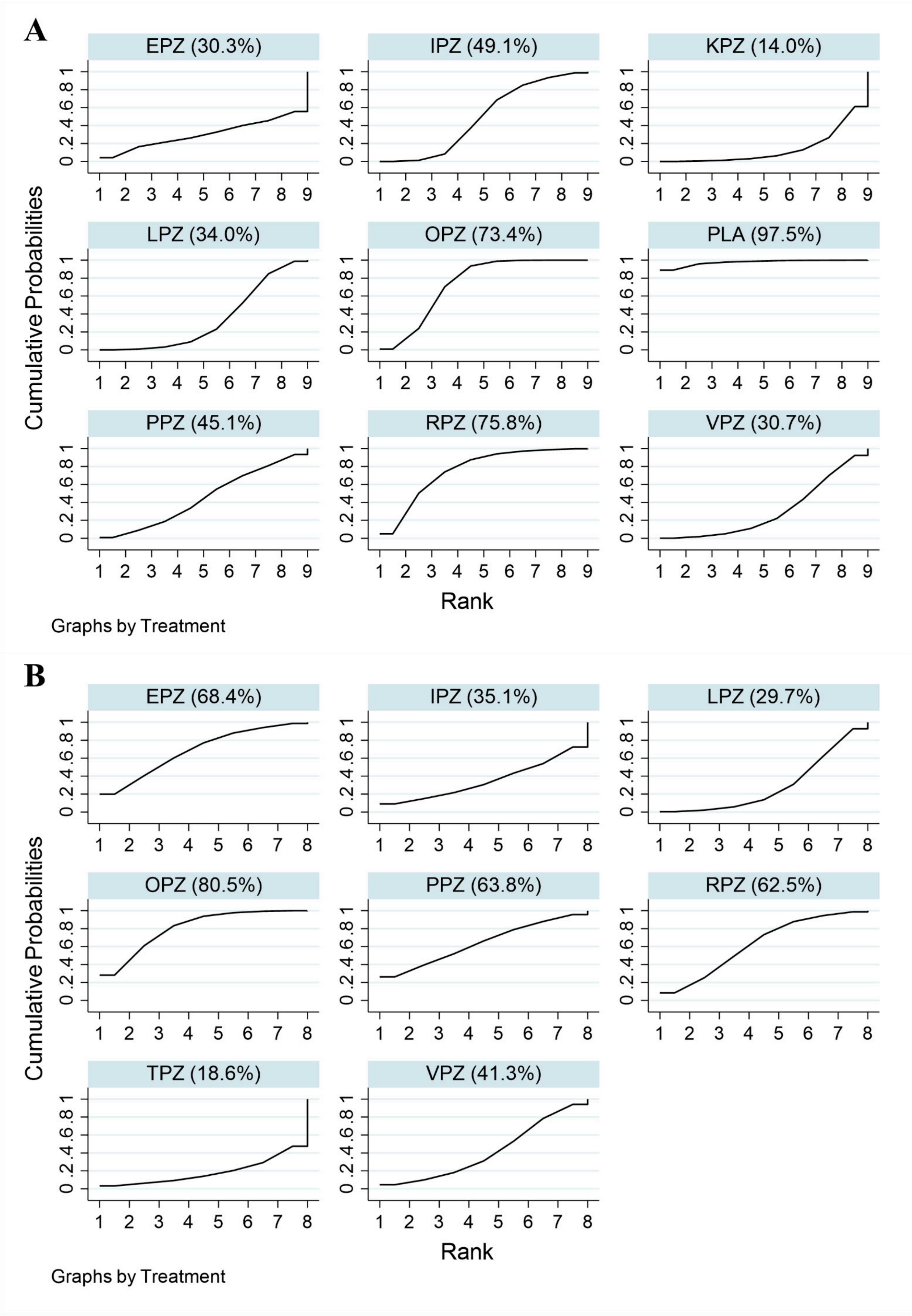

Figure S19. Forest plot of meta-analysis results for drug-related adverse events.

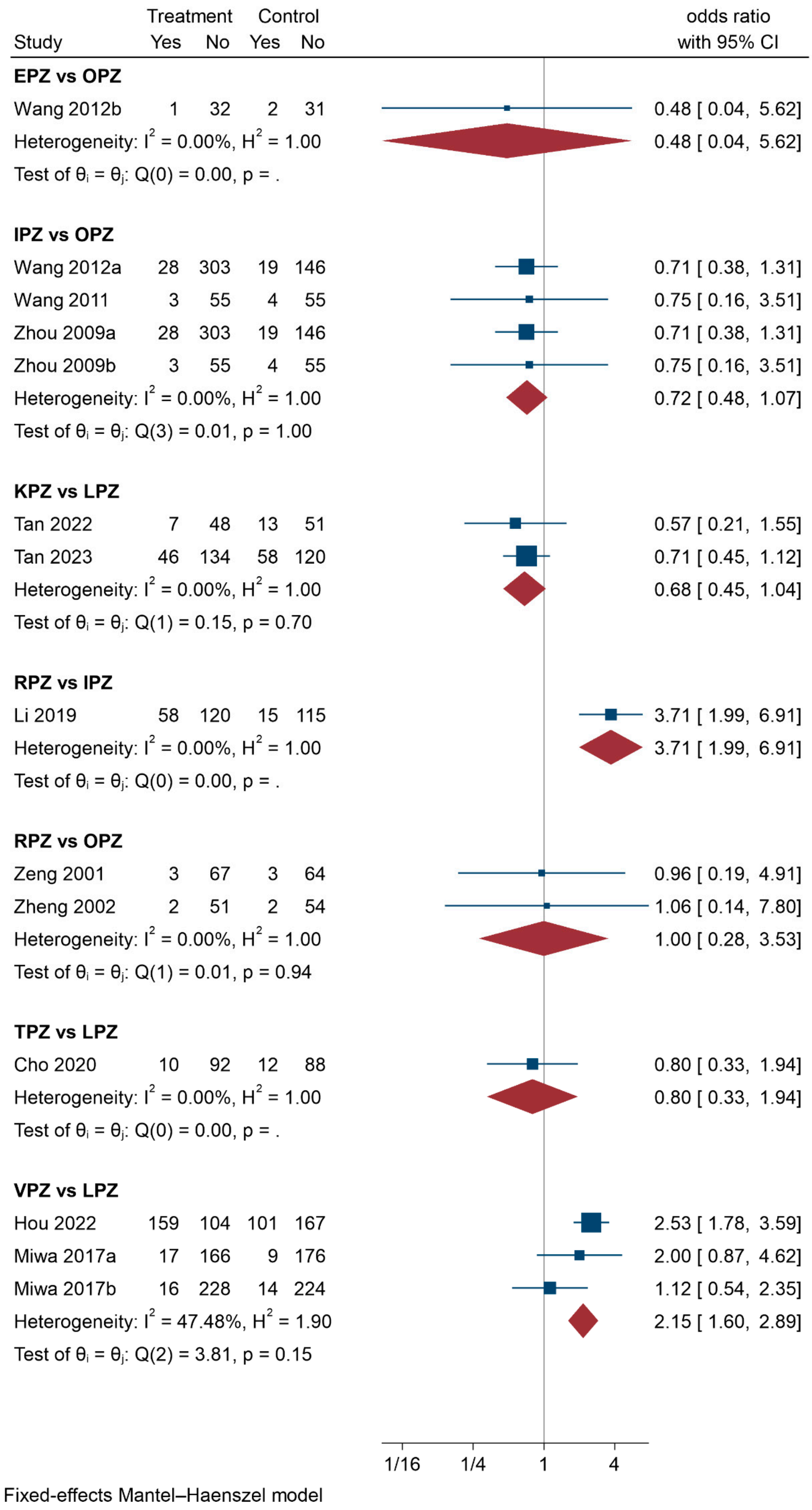

Figure S20. Forest plot of meta-analysis results for serious adverse events.

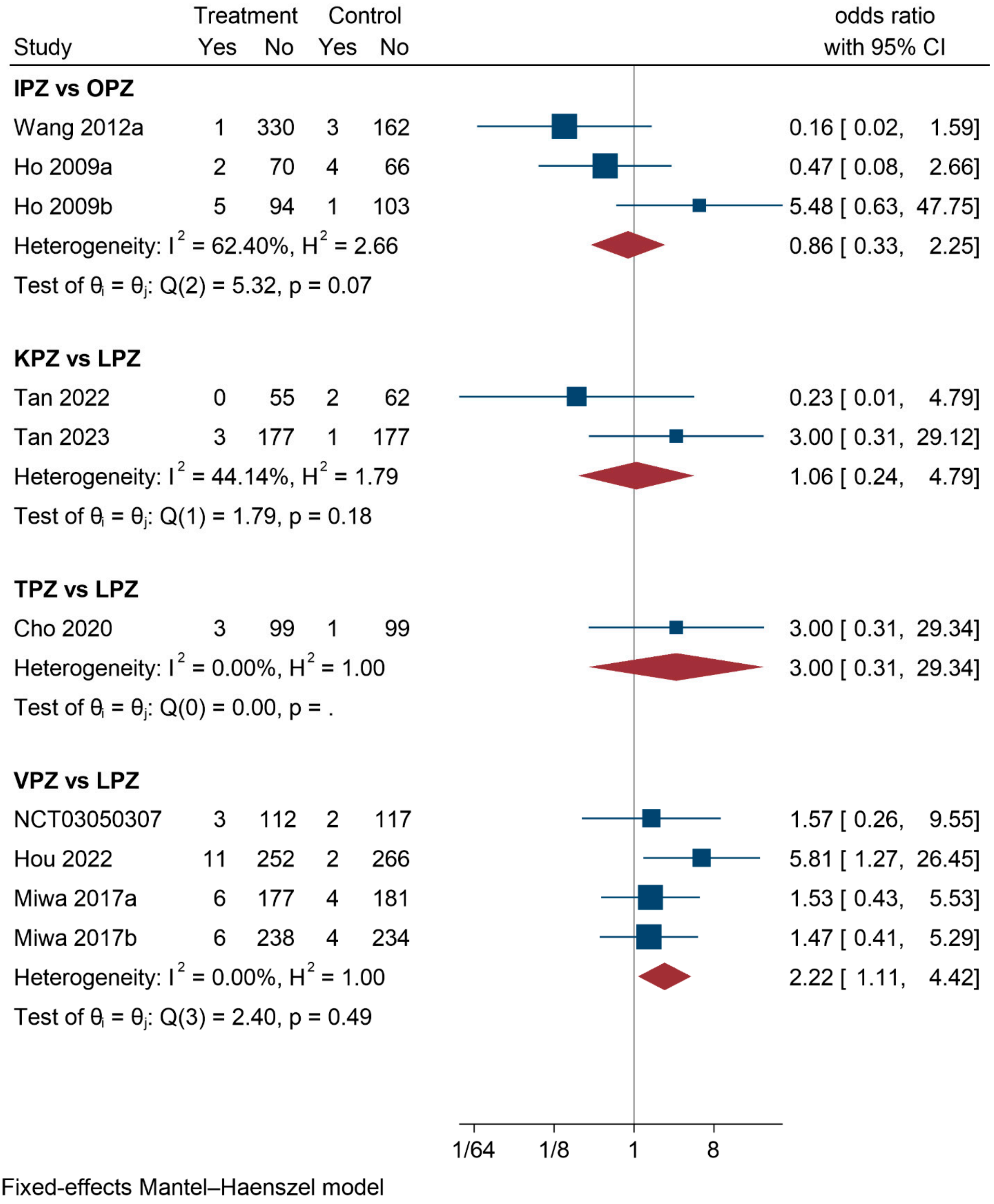

Figure S21. The results of SUCRA for different treatment regimens from the network meta-analysis. (A) SUCRA results of ulcer healing rates and *H. pylori* eradication rates; (B) SUCRA results of ulcer healing rates and pain symptom remission rates; (C) SUCRA results of ulcer healing rates and adverse events; SUCRA: the surface under the cumulative ranking.

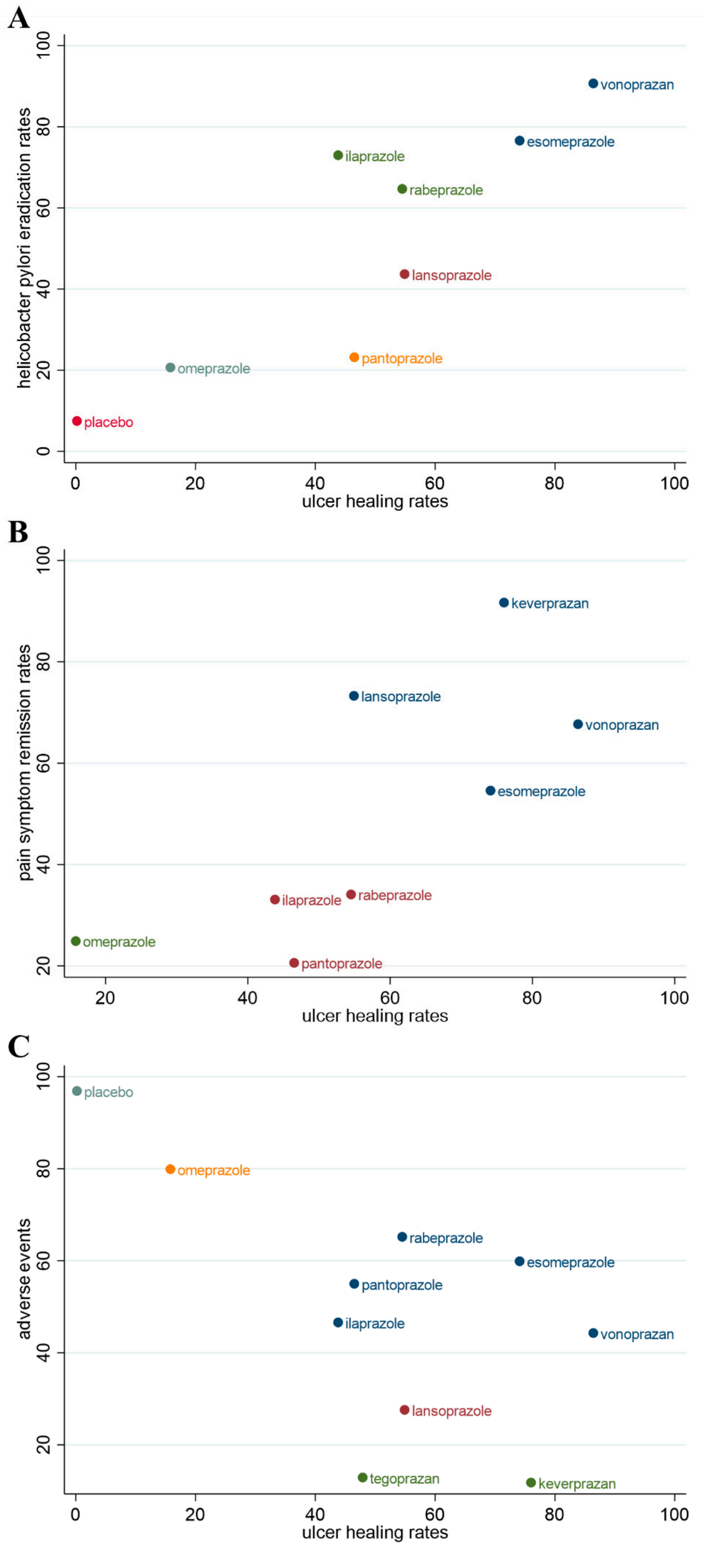

Figure S22. Forest plot of meta-analysis results for ulcer healing rate according to *Helicobacter pylori*-negative peptic ulcer patients.

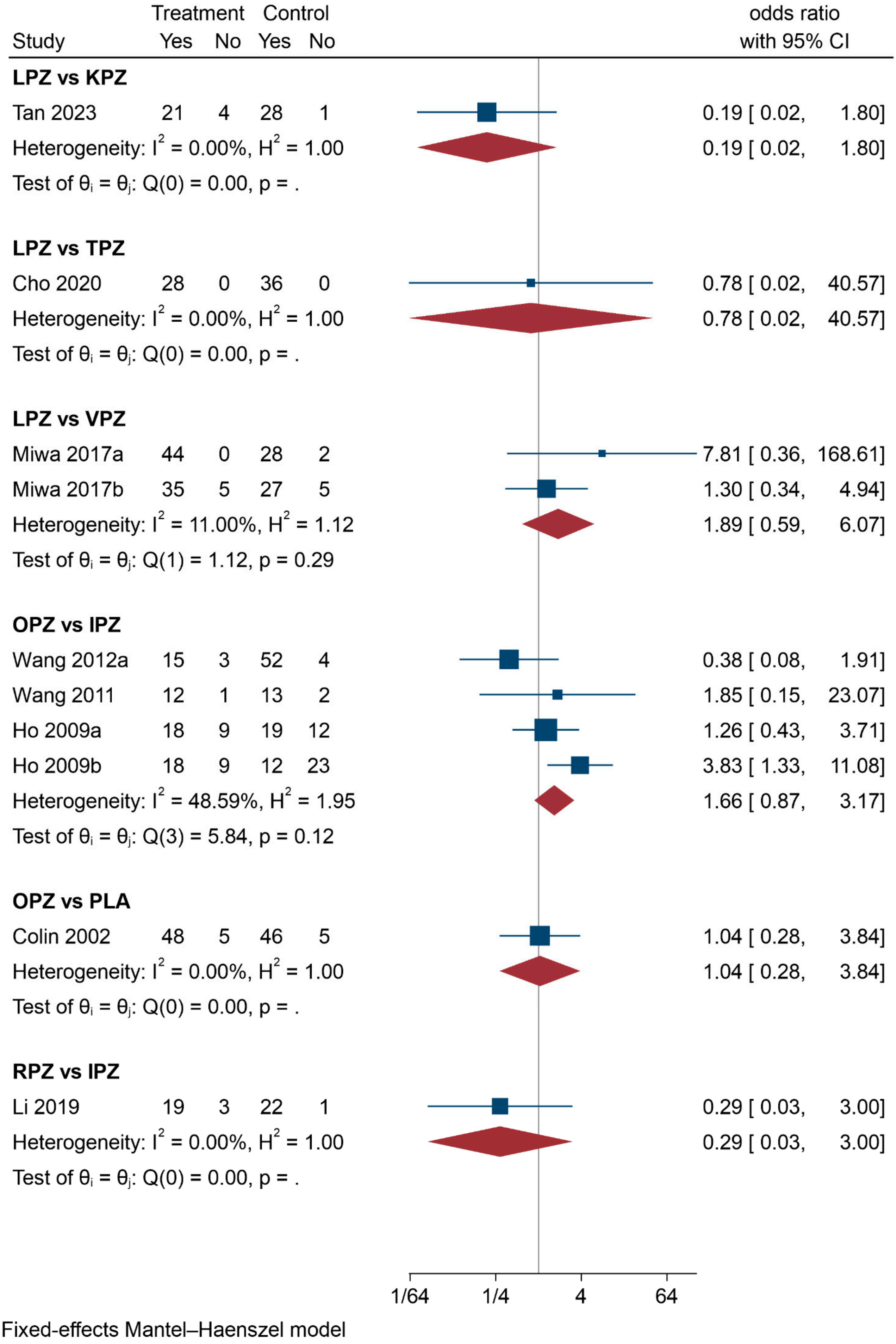

Figure S23. Forest plot of meta-analysis results for 8-week ulcer healing rate.

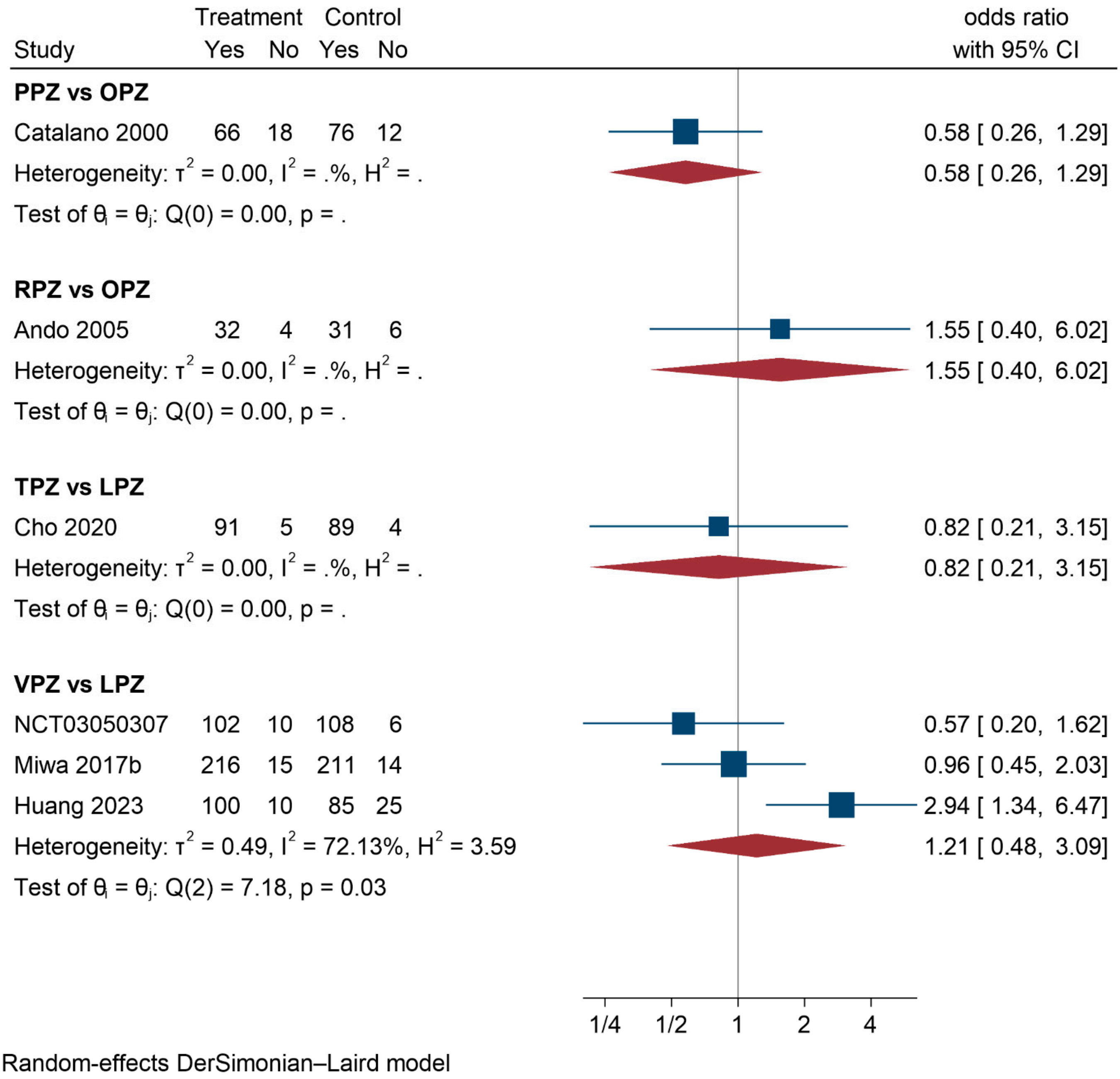

References

1. Hou, X.; Meng, F.; Wang, J.; Sha, W.; Chiu, C.-T.; Chung, W.C.; Gu, L.; Kudou, K.; Chong, C.F.; Zhang, S. Vonoprazan Non-Inferior to Lansoprazole in Treating Duodenal Ulcer and Eradicating Helicobacter Pylori in Asian Patients. *Journal of Gastroenterology and Hepatology* **2022**, *37*, 1275–1283, doi:10.1111/jgh.15837.
2. Miwa, H.; Uedo, N.; Watari, J.; Mori, Y.; Sakurai, Y.; Takanami, Y.; Nishimura, A.; Tatsumi, T.; Sakaki, N. Randomised Clinical Trial: Efficacy and Safety of Vonoprazan vs. Lansoprazole in Patients with Gastric or Duodenal Ulcers – Results from Two Phase 3, Non-inferiority Randomised Controlled Trials. *Aliment Pharmacol Ther* **2017**, *45*, 240–252, doi:10.1111/apt.13876.
3. Tan, N.; Liu, X.; Liu, C.; Li, S.; Chen, H.; Li, X.; Wu, H.; Liao, A.-J.; Zhen, Y.; Shen, P.; et al. Efficacy of Keverprazan for Duodenal Ulcer: A Phase II Randomized, Double-Blind, Parallel-Controlled Trial. *Journal of Gastroenterology and Hepatology* **2022**, *37*, 2060–2066, doi:10.1111/jgh.16000.
4. Tan, N.; Miao, X.; Liao, A.; Liu, C.; Wu, H.; Chen, H.; Li, F.; Guo, Q.; Li, S.; Tang, Y.; et al. Efficacy and Safety of Keverprazan Compared With Lansoprazole in the Treatment of Duodenal Ulcer: A Phase III, Randomized, Double-Blind, Multicenter Trial. *Clin Transl Gastroenterol* **2023**, *14*, e00602, doi:10.14309/ctg.0000000000000602.
5. Avner, D.L.; Dorsch, E.R.; Jennings, D.E.; Greski-Rose, P.A. A Comparison of Three Doses of Lansoprazole (15, 30 and 60 Mg) and Placebo in the Treatment of Duodenal Ulcer. The Lansoprazole Study Group. *Aliment Pharmacol Ther* **1995**, *9*, 521–528, doi:10.1111/j.1365-2036.1995.tb00415.x.
6. Lanza, F.; Goff, J.; Scowcroft, C.; Jennings, D.; Greski-Rose, P. Double-Blind Comparison of Lansoprazole, Ranitidine, and Placebo in the Treatment of Acute Duodenal Ulcer. Lansoprazole Study Group. *Am J Gastroenterol* **1994**, *89*, 1191–1200.
7. Graham, D.Y.; McCullough, A.; Sklar, M.; Sontag, S.J.; Roufail, W.M.; Stone, R.C.; Bishop, R.H.; Gitlin, N.; Cagliola, A.J.; Berman, R.S. Omeprazole versus Placebo in Duodenal Ulcer Healing. The United States Experience. *Dig Dis Sci* **1990**, *35*, 66–72, doi:10.1007/BF01537225.
8. Colin, R.; Hepylog Investigator Study Group. Duodenal Ulcer Healing with 1-Week Eradication Triple Therapy Followed, or Not, by Anti-Secretory Treatment: A Multicentre Double-Blind Placebo-Controlled Trial. *Aliment Pharmacol Ther* **2002**, *16*, 1157–1162, doi:10.1046/j.1365-2036.2002.01260.x.
9. Catalano, F.; Catanzaro, R.; Branciforte, G.; Bentivegna, C.; Cipolla, R.; Brogna, A.; Nuciforo, G. Five-Day Triple Therapy in Helicobacter Pylori-Positive Duodenal Ulcer: An Eighteen-Month Follow-Up. *J Clin Gastroenterol* **2000**, *31*, 130–136, doi:10.1097/00004836-200009000-00008.
10. Chang, F.-Y.; Chiang, C.-Y.; Tam, T.-N.; NG, W.-W.; Lee, S.-D. Comparison of Lansoprazole and Omeprazole in the Short-Term Management of Duodenal Ulcers in Taiwan. *Journal of gastroenterology and hepatology* **1995**, *10*, 595–601, doi:10.1111/j.1440-1746.1995.tb01352.x.
11. Li, F.; Qin, X.; Wang, L.; Han, Y.; Xia, J.; Hu, H. Ilaprazole Compared With Rabeprazole in the Treatment of Duodenal Ulcer: A Randomized, Double-Blind, Active-Controlled, Multicenter Study. *J Clin Gastroenterol* **2019**, *53*, 641–647, doi:10.1097/MCG.0000000000001186.
12. Wang, L.; Zhou, L.; Hu, H.; Lin, S.; Xia, J. Ilaprazole for the Treatment of Duodenal Ulcer: A Randomized, Double-Blind and Controlled Phase III Trial. *Curr Med Res Opin* **2012**, *28*, 101–109, doi:10.1185/03007995.2011.639353.
13. Wang, L.; Zhou, L.; Lin, S.; Hu, H.; Xia, J. A New PPI, Ilaprazole Compared with Omeprazole in the Treatment of Duodenal Ulcer: A Randomized Double-Blind Multicenter Trial. *J Clin Gastroenterol* **2011**, *45*, 322–329, doi:10.1097/MCG.0b013e3181e88515.
14. Rehner, M.; Rohner, H.G.; Schepp, W. Comparison of Pantoprazole versus Omeprazole in the Treatment of Acute Duodenal Ulceration—a Multicentre Study. *Alimentary Pharmacology & Therapeutics* **1995**, *9*, 411–416, doi:10.1111/j.1365-2036.1995.tb00399.x.
15. Ekström, P.; Carling, L.; Unge, P.; Anker-Hansen, O.; Sjöstedt, S.; Sellström, H. Lansoprazole versus Omeprazole in Active Duodenal Ulcer a Double-Blind, Randomized, Comparative Study. *Scandinavian Journal of Gastroenterology* **1995**, *30*, 210–215, doi:10.3109/00365529509093265.
16. Dekkers, C.P.; Beker, J.A.; Thjodleifsson, B.; Gabryelewicz, A.; Bell, N.E.; Humphries, T.J. Comparison of Rabeprazole 20 Mg versus Omeprazole 20 Mg in the Treatment of Active Duodenal Ulcer: A European Multicentre Study. *Aliment Pharmacol Ther* **1999**, *13*, 179–186, doi:10.1046/j.1365-2036.1999.00449.x.
17. Dobrilla, G.; Piazzi, L.; Fiocca, R. Lansoprazole versus Omeprazole for Duodenal Ulcer Healing and Prevention of Relapse: A Randomized, Multicenter, Double-Masked Trial. *Clinical Therapeutics* **1999**, *21*, 1321–1332, doi:10.1016/S0149-2918(99)80033-2.
18. Ho, K.Y.; Kuan, A.; Zaño, F.; Goh, K.L.; Mahachai, V.; Kim, D.Y.; Yoon, H.M. Randomized, Parallel, Double-Blind Comparison

of the Ulcer-Healing Effects of Ilaprazole and Omeprazole in the Treatment of Gastric and Duodenal Ulcers. *J Gastroenterol* **2009**, *44*, 697–707, doi:10.1007/s00535-009-0072-4.

19. Zhou L. Effect of ilaprazole on duodenal ulcer and the influence of CYP2C19 polymorphisms: a multicenter clinical trial. *Chinese Journal of Digestive Endoscopy* **2009**, *26*, 475–479, doi:10.3760/cma.j.issn.1007-5232.2009.09.012.
20. Zheng Q.; Wu S.; Ke M.; Liu X.; Liu N.; Dan Z. Rabeprazole-based triple therapy versus omeprazole-based triple therapy for the eradication of helicobacter pylori infection: a multicentre, randomized, double-blind, paralled-controlled study. *Chinese Journal of Gastroenterology* **2002**, *5*, 272–276.
21. Zhou L. Evaluation of ilaprazole in treatment of duodenal ulcer: a multicenter, randomized, double-blind, controlled clinical trial. *Chinese Journal of Digestion* **2009**, *29*, 542–546, doi:10.3760/cma.j.issn.0254-1432.2009.08.011.
22. Liu Y.; Wang J. A multicenter, randomized, double blind, parallel controlled study of rabeprazole and omeprazole triple therapy in helicobacter pylori eradication. *Chinese Community Doctors* **2017**, *33*, 73–74.
23. Shi Z.; Zhao F.; Chen M. Comparison of efficacy between ilaprazole and omeprazole in treatment of helicobacter pylori-associated duodenal ulcer. *Evaluation and Analysis of Drug-Use in Hospitals of China* **2019**, *19*, 1485–1487, doi:10.14009/j.issn.1672-2124.2019.12.020.
24. Deng L.; Wu M.; Lao H.; Yang H. Observation on the curative effect of PPI triple esomeprazole schemes in the treatment of duodenal ulcer. *HeBei Medicine* **2009**, *15*, 186–188.
25. Zou J. Comparison of two proton pump inhibitors in the treatment of Helicobacter pylori-positive duodenal ulcer. *Contemporary Medicine* **2014**, *20*, 146–147.
26. Fang Y. Comparative study of esomeprazole versus omeprazole in patients with duodenal ulcer. *The Medical Forum* **2016**, *20*, 445–447, doi:10.19435/j.1672-1721.2016.04.007.
27. Xia Z. Efficacy of esomeprazole in the treatment of Helicobacter pylori-positive duodenal bulb ulcers. *Guide of China Medicine* **2014**, *12*, 201–202, doi:10.15912/j.cnki.gocm.2014.19.149.
28. Zhang T. Efficacy of esomeprazole triple therapy in the treatment of duodenal bulbar ulcer. *Guide of China Medicine* **2011**, *9*, 90–91, doi:10.15912/j.cnki.gocm.2011.10.072.
29. Takeda A Randomized Double-Blind, Double-Dummy, Phase 3 Study to Evaluate the Efficacy and Safety of Oral TAK-438 20mg Compared to Lansoprazole 30mg Once- or Twice-Daily in the Treatment of Endoscopically Confirmed Gastric Ulcer Subjects With or Without Helicobacter Pylori Infection Available online: https://clinicaltrials.gov/study/NCT03050307 (accessed on 1 January 2024).
30. Cho, Y.K.; Choi, M.-G.; Choi, S.C.; Lee, K.M.; Kim, T.O.; Park, S.-H.; Moon, J.S.; Lim, Y.J.; Kang, D.H.; Cheon, G.J.; et al. Randomised Clinical Trial: Tegoprazan, a Novel Potassium-Competitive Acid Blocker, or Lansoprazole in the Treatment of Gastric Ulcer. *Aliment Pharmacol Ther* **2020**, *52*, 789–797, doi:10.1111/apt.15865.
31. Ando, T.; Kato, H.; Sugimoto, N.; Nagao, Y.; Seto, N.; Hongo, H.; Kajikawa, H.; Isozaki, Y.; Shimozawa, M.; Naito, Y.; et al. A Comparative Study on Endoscopic Ulcer Healing of Omeprazole versus Rabeprazole with Respect to CYP2C19 Genotypic Differences. *Dig Dis Sci* **2005**, *50*, 1625–1631, doi:10.1007/s10620-005-2907-z.
32. Chen, J. Efficacy and safety of vonoprazan and amoxicillin for the treatment of Helicobacter pylori-infected gastric ulcer. *China Practical Medical* **2021**, *16*, 125–127, doi:10.14163/j.cnki.11-5547/r.2021.01.052.
33. Xiao J. Pharmacoeconomics evaluation on 2 proton pump inhibitors in treatment of active stomach ulcer accompanied by hp infection. *HeBei Medicine* **2014**, *20*, 413–415.
34. Chen X.; Wei Y.; Zhong J. Clinical analysis of three common proton pump inhibitors in the treatment of gastric ulcer. *China Licensed Pharmacist* **2018**, *15*, 28–31.
35. Kong X. Clinical comparison of amoxicillin combined with two proton pump inhibitors in the treatment of gastric ulcer. *Psychologies Magazine* **2019**, *14*, 107–108, doi:10.19738/j.cnki.psy.2019.09.093.
36. Liao Q.; Zhu J. Cost-effectiveness analysis of several commonly used proton pump inhibitors in the treatment of gastric ulcer. *Strait Pharmaceutical Journal* **2007**, *10*, 110–111.
37. Hawkey, C.J.; Atherton, J.C.; Treichel, H.C.; Thjodleifsson, B.; Ravic, M. Safety and Efficacy of 7-Day Rabeprazole- and Omeprazole-Based Triple Therapy Regimens for the Eradication of Helicobacter Pylori in Patients with Documented Peptic Ulcer Disease. *Aliment Pharmacol Ther* **2003**, *17*, 1065–1074, doi:10.1046/j.1365-2036.2003.01492.x.
38. Ji, S.; Kim, H.S.; Kim, J.W.; Jee, M.K.; Park, K.W.; Uh, Y.; Lee, D.K.; Song, J.S.; Baik, S.K.; Kwon, S.O. Comparison of the Efficacy of Rabeprazole 10 Mg and Omeprazole 20 Mg for the Healing Rapidity of Peptic Ulcer Diseases. *Journal of Gastroenterology and*

Hepatology **2006**, *21*, 1381–1387, doi:10.1111/j.1440-1746.2006.04314.x.
39. Spinzi, G.C.; Bierti, L.; Bortoli, A.; Colombo, E.; Fertitta, A.M.; Lanzi, G.L.; Venturelli, R.; Minoli, G. Comparison of Omeprazole and Lansoprazole in Short-Term Triple Therapy for Helicobacter Pylori Infection. *Aliment Pharmacol Ther* **1998**, *12*, 433–438, doi:10.1046/j.1365-2036.1998.00319.x.
40. Zeng M. Multi-center clinical study of rabeprazole in the treatment of peptic ulcer. *Chinese Journal of Digestion* **2001**, *7*, 16–18.
41. Lin G.; Xiao S.; Zhang D.; Jiang S.; Yuan Y.; Xu G. Pantoprazole in the treatment of active peptic ulcer. *Chinese Journal of Digestion* **2001**, *1*, 18–20.
42. Liu F. Therapeutic effect of pantoprazole on 50 cases of peptic ulcer. *Chinese Journal of Practical Medicine* **2005**, *7*, 15–16.
43. Feng, L.-Y.; Yao, X.-X.; Jiang, S.-L. Effects of Killing Helicobacter Pylori Quadruple Therapy on Peptic Ulcer: A Randomized Double-Blind Clinical Trial. *World J Gastroenterol* **2005**, *11*, 1083–1086, doi:10.3748/wjg.v11.i7.1083.
44. Wang P.; Pan A.; Luo X.; Guo R. Efficacy and safety of vonoprazan combined with amoxicillin and clarithromycin in the treatment of peptic ulcer infected with Helicobacter pylori. *Journal of Clinical Research* **2022**, *39*, 785–787, doi:10.3969/j.issn.1671-7171.2022.05.042.
45. Huang W. A study on the efficacy, safety, recurrence rate and quality of life of vonoprazan in Helicobacter pylori-positive peptic ulcer patients. *Heilongjiang Medicine Journal* **2023**, *36*, 1102–1105, doi:10.14035/j.cnki.hljyy.2023.05.037.
46. Qin, Y.; Chen, P. Effect of Vonoprazan Combined with Amoxicillin and Clarithromycin in the Treatment of Peptic Ulcer Infected by Helicobacter Pylori. *Chinese and Foreign Medical Research* **2023**, *21*, 48–51, doi:10.14033/j.cnki.cfmr.2023.21.012.
47. Yu Q.; Li C. Effect of vonoprazan combined with amoxicillin in the treatment of HP-positive peptic ulcer and its effect on inflammatory response and gastrointestinal function. *Chinese Journal of Clinical Rational Drug Use* **2023**, *16*, 71–74, doi:10.15887/j.cnki.13-1389/r.2023.36.020.
48. Chen C. Clinical efficacy of different proton pump inhibitors in combination with clarithromycin and amoxicillin in the treatment of Helicobacter pylori (Hp)-positive peptic ulcers. *Health for Everyone* **2020**, *14*, 663.
49. Zhang Y. Treating Hp-related digestive ulcer with esomeprazole. *Chinese Journal of Primary Medicine and Pharmacy* **2007**, *14*, 1962–1963.
50. Sun H.; Zhang X. The clinical observation of rabeprazole triple therapy for peptic ulcer. *Chinese Journal of Clinical Rational Drug Use* **2011**, *4*, 25–26, doi:10.15887/j.cnki.13-1389/r.2011.21.073.
51. Li H.; Fan H. Comparative analysis of the efficacy of different proton pump inhibitors combined with clarithromycin and amoxicillin in the treatment of Hp-positive peptic ulcer. *Chinese Journal of Modern Drug Application* **2018**, *12*, 130–131, doi:10.14164/j.cnki.cn11-5581/r.2018.07.073.
52. Wang H.; Tan X.; Dong X. Comparison of the efficacy of esomeprazole and omeprazole in the treatment of peptic ulcer. *Strait Pharmaceutical Journal* **2012**, *24*, 146–147.
53. Li Y. Clinical observation of rabeprazole in the treatment of Helicobacter pylori-positive peptic ulcer. *China Medical Herald* **2009**, *6*, 58–59.
54. Yang J.; Xu C.; Zhou L.; Wang P. Curative effect of lansoprazole on peptic ulcer. *China Pharmaceuticals* **2000**, *9*, 41–42, doi:DOI:10.3969/j.issn.1006-4931.2000.04.045.
